# The gut metabolite indole-3-propionic acid activates ERK1 to restore social function and hippocampal inhibitory synaptic transmission in a 16p11.2 microdeletion mouse model

**DOI:** 10.1186/s40168-024-01755-7

**Published:** 2024-03-28

**Authors:** Jian Jiang, Dilong Wang, Youheng Jiang, Xiuyan Yang, Runfeng Sun, Jinlong Chang, Wenhui Zhu, Peijia Yao, Kun Song, Shuwen Chang, Hong Wang, Lei Zhou, Xue-Song Zhang, Huiliang Li, Ningning Li

**Affiliations:** 1 Tomas Lindahl Nobel Laureate Laboratory, The Seventh Affiliated Hospital, Sun Yat-Sen University, Shenzhen, China; 2 Department of Pediatrics, Sun Yat-Sen Memorial Hospital, Sun Yat-Sen University, Guangzhou, China; 3 Institute of Molecular Physiology, Shenzhen Bay Laboratory, Shenzhen, China; 4 Brain Research Centre, Department of Biology, School of Life Sciences, Southern University of Science and Technology, Shenzhen, China; 5 The Brain Cognition and Brain Disease Institute (BCBDI), Shenzhen-Hong Kong Institute of Brain Science Shenzhen Fundamental Research Institutions, Shenzhen Institute of Advanced Technology, Chinese Academy of Sciences, Shenzhen, China; 6 Center for Advanced Biotechnology and Medicine, Rutgers University, Piscataway, NJ USA; 7 Wolfson Institute for Biomedical Research, Division of Medicine, Faculty of Medical Sciences, University College London, London, UK; 8 China-UK Institute for Frontier Science, Shenzhen, China; 9 Department of Anesthesiology, The Afliated Hospital of Youjiang Medical University for Nationalities, Baise, China

**Keywords:** Autism, Social deficits, Gut microbiota metabolite, Indole-3-propionic acid, Mapk3, GABA

## Abstract

**Background:**

Microdeletion of the human chromosomal region 16p11.2 (16p11.2${}^{+/-}$) is a prevalent genetic factor associated with autism spectrum disorder (ASD) and other neurodevelopmental disorders. However its pathogenic mechanism remains unclear, and effective treatments for 16p11.2${}^{+/-}$ syndrome are lacking. Emerging evidence suggests that the gut microbiota and its metabolites are inextricably linked to host behavior through the gut-brain axis and are therefore implicated in ASD development. Despite this, the functional roles of microbial metabolites in the context of 16p11.2${}^{+/-}$ are yet to be elucidated. This study aims to investigate the therapeutic potential of indole-3-propionic acid (IPA), a gut microbiota metabolite, in addressing behavioral and neural deficits associated with 16p11.2${}^{+/-}$, as well as the underlying molecular mechanisms.

**Results:**

Mice with the 16p11.2${}^{+/-}$ showed dysbiosis of the gut microbiota and a significant decrease in IPA levels in feces and blood circulation. Further, these mice exhibited significant social and cognitive memory impairments, along with hyperactivation of hippocampal dentate gyrus neurons and reduced inhibitory synaptic transmission in this region. However, oral administration of IPA effectively mitigated the histological and electrophysiological alterations, thereby ameliorating the social and cognitive deficits of the mice. Remarkably, IPA treatment significantly increased the phosphorylation level of ERK1, a protein encoded by the *Mapk3* gene in the 16p11.2 region, without affecting the transcription and translation of the *Mapk3* gene.

**Conclusions:**

Our study reveals that 16p11.2${}^{+/-}$ leads to a decline in gut metabolite IPA levels; however, IPA supplementation notably reverses the behavioral and neural phenotypes of 16p11.2${}^{+/-}$ mice. These findings provide new insights into the critical role of gut microbial metabolites in ASD pathogenesis and present a promising treatment strategy for social and cognitive memory deficit disorders, such as 16p11.2 microdeletion syndrome.

Video Abstract

**Supplementary Information:**

The online version contains supplementary material available at 10.1186/s40168-024-01755-7.

## Background

Autism spectrum disorder (ASD) is a group of early neurodevelopmental disorders, characterized by social deficits, repetitive behaviors and stereotyped interests [1]. The incidence of ASD worldwide has been reported to be as high as 1% and is steadily increasing [2]. While both genetic and environmental cues are recognized as the crucial risk factors for ASD [1], its pathogenesis remains largely unclear. Chromosomal copy number variations (CNVs) are present in 5-10% of individuals with ASD [3], and the most common CNV found in ASD is the human chromosome 16p11.2 microdeletion (16p11.2${}^{+/-}$), which results in developmental delay, impaired communication and intellectual disability, being responsible for 1% of all ASD [4]. This microdeletion spans approximately 600 kb and encompasses 27-29 genes [5, 6], including *MAPK3* [7], *KCTD13* [8], and *TAOK2* [9], all of which have been implicated in the pathogenesis of ASD. Exhilaratingly, the 16p11.2${}^{+/-}$ phenotype has been sophisticatedly modeled in mice, which exhibit neurodevelopmental, social, and cognitive deficits that phenocopy human autistic features [10, 11]. Therefore, 16p11.2${}^{+/-}$ mice are considered a reliable tool for uncovering the causal factors underlying the atypical social brain of ASD.

Emerging evidence indicates that dysbiosis of the gut microbiome (GM) may play a pivotal role in ASD pathogenesis through the potential microbiome-gut-brain axis. Germ-free mice lacking typical GM have been shown to display significant social impairments [12]. GM dysbiosis has also been observed in some known ASD mouse models, such as *Shank3* mutant mice [13, 14] and BTBR mice [15]. A recent report has even suggested that gut bacteria from individuals with ASD can directly cause ASD-like behaviors in mice [16]. While concerns exist that dietary preferences may influence the GM in ASD individuals and thus affect the development of the disorder [17], an intrinsic link between ASD and GM is well recognized. Investigating the association between disease and gut microbiota, as well as between the host and gut metabolites, in animal models can help eliminate confounding factors such as region and diet to a certain extent.

Thousands of small molecules and metabolites are involved in host-microbe communication [18], including in the modulation of central nervous system (CNS) signaling to affect the behaviors of patients with neuropsychiatric disorders such as anxiety, depressive disorder [19], and epilepsy [20]. Recent studies have highlighted the role of gut microbiota and its metabolites, such as glutamine [21] and vitamin B6 [22], in regulating social behaviors in ASD models associated with Chd8 and EphB6 deficiencies by disturbing the excitatory-inhibitory (E-I) balance of synapse transmission. An E-I ratio imbalance has also been observed in the cerebral cortex of the 16p11.2${}^{+/-}$ mouse model [23], but the contribution of GM and its metabolic mediators to the 16p11.2${}^{+/-}$ social impairments remains unknown. Gut microbial metabolism is exemplified by a range of small molecules/metabolites, including tryptophan metabolites and short-chain fatty acids that can accumulate in the gut or reach distant organs, influencing a variety of physiological functions such as immune cell responses and neuronal excitability [24]. Among these metabolites, indole-3-propionic acid (IPA), a tryptophan metabolite exclusively produced by the microbiota, has shown a potential neuroprotective effect on CNS diseases such as Alzheimer’s disease and diabetes-induced cognitive impairment [25–27]. In addition, recent studies have shown that IPA facilitates axonal regeneration and functional recovery by inhibition of neutrophil chemotaxis in the peripheral nervous system [28]. Despite its broad involvement, the effect of IPA on social behavior remains largely unknown.

Our study on 16p11.2${}^{+/-}$ mice revealed a significant disturbance in their GM, which was accompanied by deficits in social novelty and recognition memory. Further, we identified an impairment in inhibitory synaptic transmission in the hippocampus of these mice. Importantly, our findings suggest that activating the MAPK3 signaling pathway via the use of the compound IPA can ameliorate the hippocampal inhibitory synaptic transmission imbalance and the social deficits in these mice. These results may have significant implications for the development of novel therapeutic strategy approaches to improve social behaviors in individuals with ASD or 16p11.2${}^{+/-}$ syndrome.

## Results

### 16p11.2${}^{+/-}$ mice showed disturbances in gut microbiota characterized by a decrease in the synthesis and circulation of IPA

**Fig. 1:**
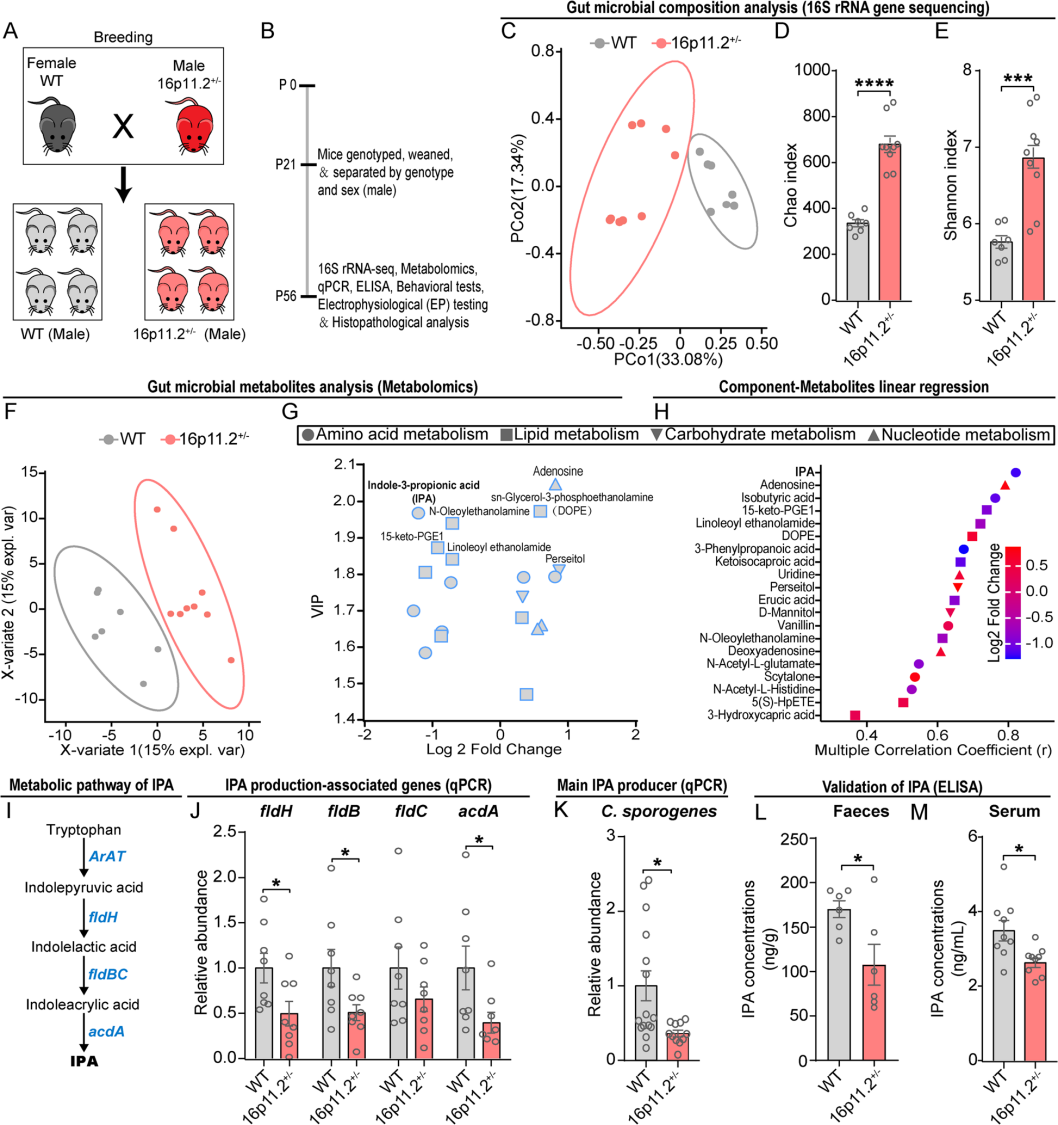
16p11.2${}^{+/-}$ mice exhibited altered microbial composition and declined synthesis and circulation of IPA. **A** The mice breeding strategies. **B** Schematic diagram for experimental design. **C**–**E** 16S rRNA gene sequencing of gut microbiota of 8-week-old WT and 16p11.2${}^{+/-}$ mice. (**C**) Principal coordinate analysis (PCoA) plot from feces of two groups (WT: *n* = 7 mice; 16p11.2${}^{+/-}$: *n* = 9 mice). α-diversity was measured by Chao (**D**) and Shannon (**E**) indexes. **F**–**H** Untargeted metabolomics was performed on feces of 8-week-old WT and 16p11.2${}^{+/-}$ mice. (**F**) Orthogonal partial least squares discriminant analysis (OPLS-DA) were used to reflect the differences between metabolites in the WT and 16p11.2${}^{+/-}$ groups. (**G**) The metabolites with significant differences were screened. (**H**) Multiple correlation coefficients suggested that IPA was most associated with structural dysbiosis (WT: *n* = 7 mice; 16p11.2${}^{+/-}$: *n* = 9 mice). **I**, **J** The expression levels of several genes associated with IPA production were decreased in 16p11.2${}^{+/-}$ mice. (**I**) Schematic representation of tryptophan metabolism leading to IPA. (**J**) The expression levels of *fldB*, *fldH*, and *acdA* were reduced in 16p11.2${}^{+/-}$ mice (WT: *n* = 8 mice; 16p11.2${}^{+/-}$: *n* = 8 mice). **K** qPCR analysis showed the levels of *C. sporogenes*, the main producer of IPA, were decreased in 16p11.2${}^{+/-}$ mice (WT: *n* = 15 mice; 16p11.2${}^{+/-}$: *n* = 11 mice). **L** The level of IPA in feces of mice was detected (WT: *n* = 6 mice; 16p11.2${}^{+/-}$: *n* = 6 mice). **M** The level of IPA in serum of mice was detected (WT: *n* = 9 mice; 16p11.2${}^{+/-}$: *n* = 9 mice)**.** Data are presented as mean ± SEM, and Student’s *t* test was applied. *p < 0.05, ***p < 0.001, ****p < 0.0001. Detailed statistical information is presented in Additional file 2: Table S1

Gastrointestinal (GI) symptoms and disorders frequently co-occur with ASD [29], and studies have shown that ASD patients commonly exhibit disturbances in their intestinal microbiota and metabolites [30–32]. To investigate whether the microbiota composition and metabolite levels are altered in 16p11.2${}^{+/-}$ mice, we performed 16S rRNA gene sequencing and untargeted metabolomics analysis on fecal samples collected from both 16p11.2${}^{+/-}$ mice and their wild-type (WT) counterparts. The mouse breeding strategies and experimental design are illustrated in Fig. 1A and B. Our principal coordinate analysis (PCoA) of 16S rRNA gene sequencing based on Bray-Curtis distance demonstrated a significant difference in the composition of the microbiota of 16p11.2${}^{+/-}$ mice compared to WT mice (Fig. 1C). Furthermore, the gut microbiota in 16p11.2${}^{+/-}$ mice exhibited significantly increased richness (Chao index, Fig. 1D) and diversity (Shannon index, Fig. 1E) compared to WT mice. These results indicated that the gut microbiota of 16p11.2${}^{+/-}$ mice was structurally dysbiotic. In addition, metabolomics analysis identified and annotated a total of 404 compounds in fecal samples. To compare metabolic profiles between WT and 16p11.2${}^{+/-}$ mice, we used orthogonal partial least squares discriminant analysis (OPLS-DA) plots. We observed that 16p11.2${}^{+/-}$ mice displayed distinct microbial metabolic profiling relative to WT mice, consistent with GM structural dysbiosis (Fig. 1F). Using a screening criteria of *p* < 0.05 and variable importance for the projection (VIP) > 1.5, we identified 20 differential metabolites between the two groups (Fig. 1G). Among these differential metabolites, seven were involved in amino acid metabolism, eight in lipid metabolism, two in carbohydrate metabolism, and three in nucleotide metabolism (Additional file 1: Fig. S1).

Next, we evaluated the association between differential metabolites and GM components using linear regression analysis. We set each metabolite as a dependent variable, and the structural indexes (Chao and Shannon) as independent variables. Multiple correlation coefficients showed that IPA was most significantly associated with the 16p11.2${}^{+/-}$ GM structural dysbiosis (Fig. 1H). To establish a 16p11.2${}^{+/-}$ specific GM-metabolite axis, we validated the abundance of genes and bacteria implicated in IPA production using qPCR [33, 34]. We assessed the expression levels of genes that encode enzymes associated with IPA production, including *fldH*, *fldB*, *fldC*, and *acdA*, which are involved in the tryptophan to IPA metabolic pathway (Fig. 1I) [24]. Notably, *fldH*, *fldB*, and *acdA* were reduced in the microbial genome of 16p11.2${}^{+/-}$ mice (Fig. 1J), consistent with the decreased level of IPA. Furthermore, the relative abundance of *Clostridium sporogenes* (*C. sporogenes*), the main producer of IPA, was significantly decreased in 16p11.2${}^{+/-}$ mice compared to the WT group (Fig. 1K). These results suggest that the profound decline of IPA in 16p11.2${}^{+/-}$ mice was a result of GM dysbiosis.

As previous studies have reported that IPA is a gut microbiota-derived antioxidant with pharmacological efficacy in neuroprotection [25, 34, 35], we aimed to investigate whether disturbances in the gut microbiota of 16p11.2${}^{+/-}$ mice affected their circulatory levels of IPA. To do this, we measured both fecal and serum levels of IPA in WT and 16p11.2${}^{+/-}$ mice using an enzyme-linked immunosorbent assay (ELISA). Consistent with our metabolomics findings, we confirmed that fecal IPA levels were significantly lower in 16p11.2${}^{+/-}$ mice compared to the WT group (Fig. 1L). Remarkably, we also observed a significant decrease in serum IPA levels in 16p11.2${}^{+/-}$ mice (Fig. 1M). In a previous study, it was found that IPA in cerebrospinal fluid is derived from gut microbiota metabolism and can cross the blood-brain barrier [36]. Our findings suggest that the observed dysbiosis in the gut microbiota of 16p11.2${}^{+/-}$ mice is associated with reduced circulatory levels of IPA, which could potentially further impair social brain function.

### 16p11.2${}^{+/-}$ mice exhibited social novelty and recognition memory deficits

**Fig. 2:**
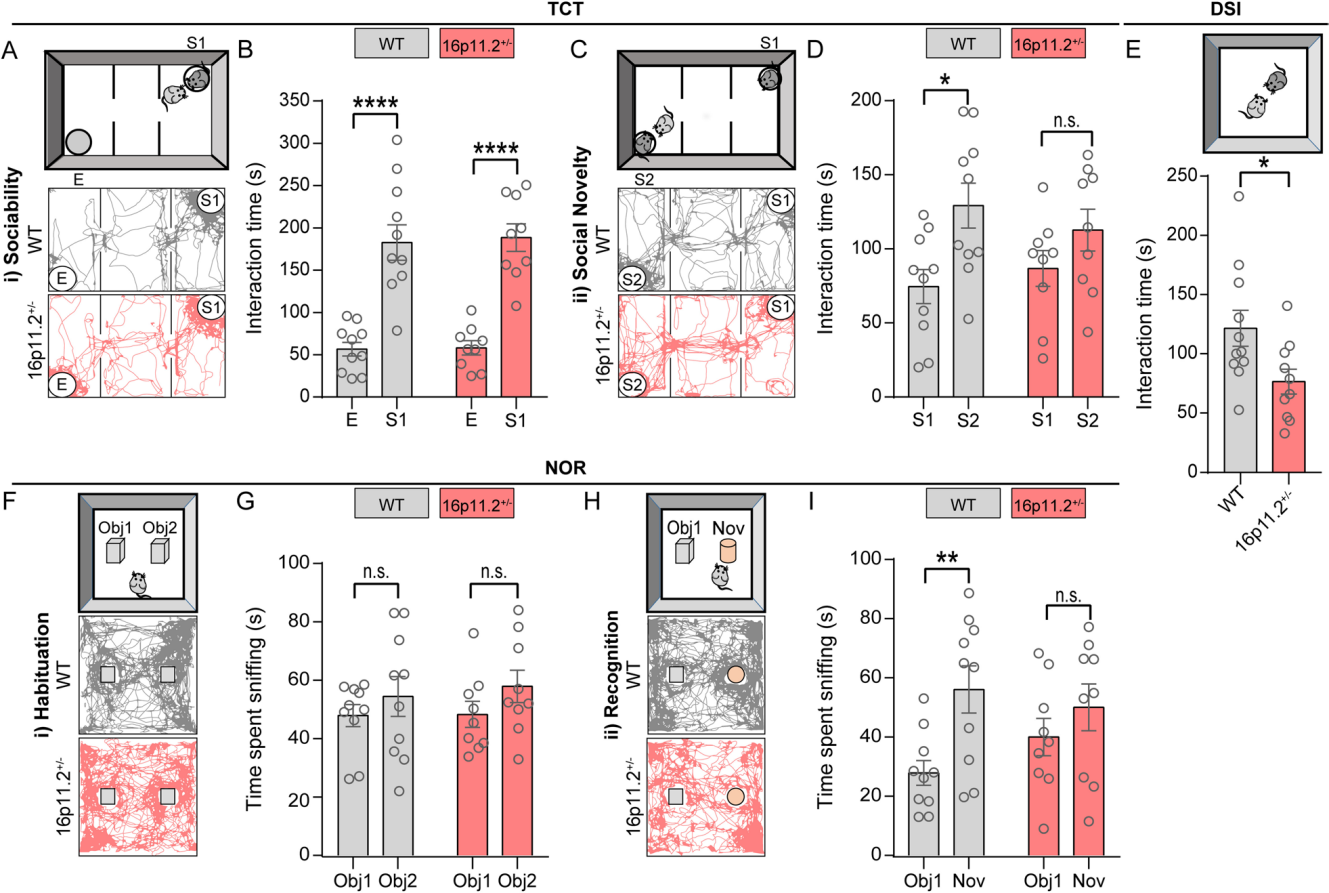
16p11.2${}^{+/-}$ mice exhibited deficits in social novelty and recognition memory comparad to WT controls. **A**-**D** Three-chamber test (TCT). (**A**) Schematic of the sociability phase of TCT (E: empty cage; S1: stranger mouse) and representative trajectories of mice. (**B**) 16p11.2${}^{+/-}$ mice exhibited comparable sociability compared to WT mice (WT: *n* = 10 mice; 16p11.2${}^{+/-}$: *n* = 9 mice. Two-way ANOVA). (**C**) Schematic of the social novelty phase of TCT (S1: stranger mouse; S2: novel stranger mouse) and representative trajectories of mice. (**D**) 16p11.2${}^{+/-}$ mice exhibited social novelty deficit (WT: *n* = 10 mice; 16p11.2${}^{+/-}$: *n* = 9 mice. Two-way ANOVA). **E** Direct social interaction (DSI) test. 16p11.2${}^{+/-}$ mice showed a significant reduction in social time compared to WT mice (WT: *n* = 11 mice; 16p11.2${}^{+/-}$: *n* = 10 mice. Student’s *t* test). **F**-**I** Novel object recognition (NOR) test. (**F**) Schematic of the habituation phase of NOR test (Obj1: object1; Obj2: object2) and representative trajectories of mice. (**G**) 16p11.2${}^{+/-}$ mice and WT mice showed no obvious preference over two identical objects (WT: *n* = 10 mice; 16p11.2${}^{+/-}$: *n* = 9 mice. Two-way ANOVA). (**H**) Schematic of the recognition phase of NOR test (Obj1: object1; Nov: novel object) and representative trajectories of mice. (**I**) 16p11.2${}^{+/-}$ mice exhibited no obvious preference over novel objects (WT: *n* = 10 mice; 16p11.2${}^{+/-}$: *n* = 9 mice. Two-way ANOVA). Data are presented as mean ± SEM. *p < 0.05, **p < 0.01, ***p < 0.001, ****p < 0.0001, and n.s.: not significant. Detailed statistical information is presented in Additional file 2: Table S1

Seeing as engagement of autistic individuals with others may be encumbered by their deficits in social understanding, we set about characterizing social and behavioral alterations in 16p11.2${}^{+/-}$ mice and teasing out specific deficits that may be linked to IPA shortage. We employed the three-chamber test (TCT), which reflects social cognition in the form of general sociability (Fig. 2A) and preference for social novelty (Fig. 2C) in rodent models of CNS disorders [37]. Similar to WT peers, 16p11.2${}^{+/-}$ mice showed a clear preference for the stranger mouse (S1) over the empty cage (E) (Fig. 2B), indicating normal sociability. However, they exhibited obvious abnormalities in social novelty, spending similar amounts of time sniffing at both the first stranger mouse (S1) and the novel stranger mouse (S2) (Fig. 2D). This outcome is consistent with a previous report suggesting social novelty deficits in 16p11.2${}^{+/-}$ mice [38]. To further dissect the social and behavioral deficit of the 16p11.2${}^{+/-}$ mice, we conducted the direct social interaction (DSI) test, which allows for a fine-grained assessment of social responses presented by a testing mouse when facing a conspecific stranger [39]. In the DSI test conducted in a neutral cage, the interaction time of 16p11.2${}^{+/-}$ mice with a stranger mouse was significantly declined than that of WT peers (Fig. 2E), indicating a social deficit in 16p11.2${}^{+/-}$ mice.

Additionally, the novel object recognition (NOR) test (Fig. 2F, H) showed that both 16p11.2${}^{+/-}$ and WT mice spent comparable time sniffing at two identical objects (Obj1, Obj2) during the habituation phase (Fig. 2G). However, the mutants showed no preference for the novel object (Nov) that was substituted for one of the familiar objects (Obj1) during the recognition phase (Fig. 2I), indicating impaired object recognition memory in the 16p11.2${}^{+/-}$ mice [40]. Consistent with a previous report [41], we found that 16p11.2${}^{+/-}$ mice exhibited significantly increased traveling distance compared to WT mice in the open field test (OFT), indicating hyperactivity (Additional file 1: Fig. S2A, B). Furthermore, we utilized an elevated plus-maze (EPM) test to evaluate anxiety-like behavior in 16p11.2${}^{+/-}$ mice. Our results indicated the absence of significant signs of anxiety-like behavior in 16p11.2${}^{+/-}$ mice (Additional file 1: Fig. S2C, D). Collectively, our behavioral tests revealed that 16p11.2${}^{+/-}$ mice exhibited autistic features dominated by impairments in social and recognition memory, as well as hyperactivity.

### Impaired inhibitory synaptic transmission in hippocampal dentate gyrus in 16p11.2${}^{+/-}$ mice

**Fig. 3:**
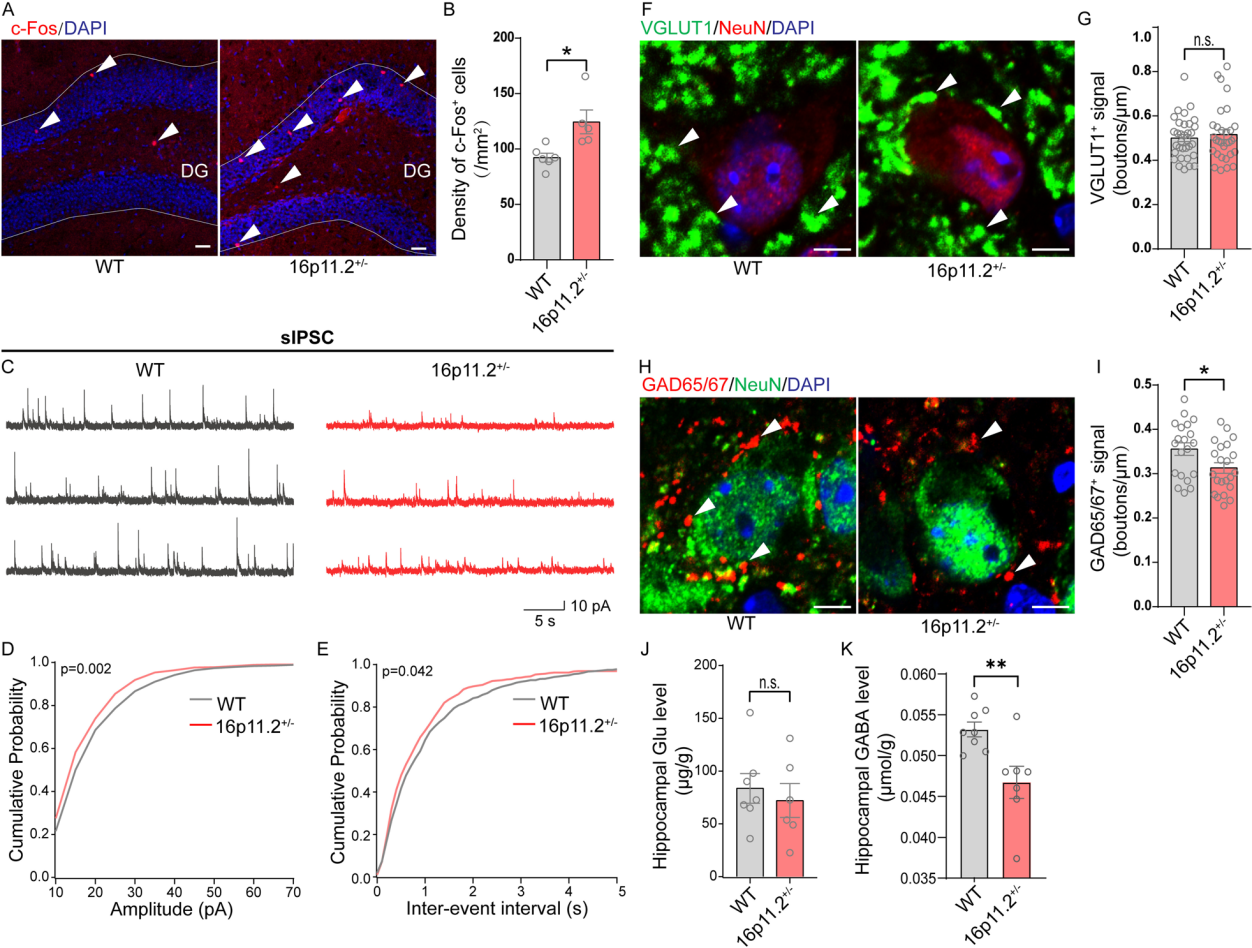
Inhibitory synaptic transmission dysfunction in the hippocampus dentate gyrus (DG) of 16p11.2${}^{+/-}$ mice. **A** Hippocampal neurons in 16p11.2${}^{+/-}$ mice were hyperactivated after the TCT. The red puncta indicate c-Fos^+^ neurons in the DG of hippocampus. Scale bar: 30 μm. **B** Quantification of the numbers of c-Fos^+^ neurons (WT: *n* = 6 mice; 16p11.2${}^{+/-}$: *n* = 5 mice. Student’s *t* test). **C** Representative sIPSCs traces from granule cells in hippocampus. Scale bars: 5 s, 10 pA. **D** Cumulative distribution of sIPSCs amplitude (WT: *n* = 914 events from 9 cells of 3 mice; 16p11.2${}^{+/-}$: *n* = 556 events from 6 cells of 5 mice. Kolmogorov-Smirnov test). **E** Cumulative distribution of sIPSCs frequency (WT: *n* = 914 events from 9 cells of 3 mice; 16p11.2${}^{+/-}$: *n* = 556 events from 6 cells of 5 mice. Kolmogorov-Smirnov test). **F** High-magnification confocal planes of VGLUT1-expressing perisomatic puncta on neurons of WT and 16p11.2${}^{+/-}$ mice. Scale bar: 5 μm. **G** Graph showing the density of VGLUT1 puncta on the perisomatic region of hippocampal neurons (WT: *n* = 33 cells from 3 mice; 16p11.2${}^{+/-}$: *n* = 31 cells from 3 mice. Student’s *t* test). **H** GAD65/67 immunoreactivity in the hippocampus of WT and 16p11.2${}^{+/-}$ mice. **I** Graph showing the density of GAD65/67 puncta on the perisomatic region of hippocampal neurons (WT: *n* = 20 cells from 3 mice; 16p11.2${}^{+/-}$: *n* = 22 cells from 3 mice. Student’s *t* test). **J** The level of glutamate (Glu) in hippocampus of mice was detected (WT: *n* = 7 mice; 16p11.2${}^{+/-}$: *n* = 6 mice. Student’s *t* test). **K** The level of GABA in hippocampus of mice was detected (WT: *n* = 8 mice; 16p11.2${}^{+/-}$: *n* = 7 mice. Student’s *t* test). Data are presented as mean ± SEM. *p < 0.05, **p < 0.01, and n.s.: not significant. Detailed statistical information is presented in Additional file 2: Table S1

The hippocampal and medial prefrontal cortex (mPFC) are critical brain regions involved in social cognition and episodic memory [42–44]. Neuroactivity abnormalities in these regions can lead to impaired social interactions [45]. To investigate potential changes in neural activity in these regions, we examined the expression of c-Fos, an immediate-early gene marker for neuronal activity, after exposing 16p11.2${}^{+/-}$ mice to social stimuli (i.e., TCT) (Fig. 3A). Intriguingly, we found a significant increase in c-Fos expression in the hippocampal dentate gyrus (DG) region of 16p11.2${}^{+/-}$ mice compared to the WT group (Fig. 3B). We also observed a trend towards increased c-Fos immunostaining in the hippocampal CA1 region (Additional file 1: Fig. S3A, B), while the density of c-Fos^+^ cells remained unchanged in the hippocampal CA3 (Additional file 1: Fig. S3C, D) and the mPFC regions (Additional file 1: Fig. S3E, F). These findings reveal that excessive neuronal activation in the hippocampus, particularly the DG region, may specifically potentiate social novelty impairment in 16p11.2${}^{+/-}$ mice.

Glutamate and γ-aminobutyric acid (GABA) are the primary excitatory and inhibitory neurotransmitters, respectively, in the CNS, and play a crucial role in regulating neuronal activity [46]. Dysfunction of neurotransmission could lead to altered neural activity patterns, which may affect the processing of social information and ultimately result in social deficits [47]. To delineate the alteration of neuroactivity and function at the level of individual neurons, we assessed the spontaneous synaptic activities of granule cells in the DG region of 16p11.2${}^{+/-}$ mice using patch-clamp recordings. We recorded postsynaptic membrane responses, including spontaneous excitatory/inhibitory postsynaptic currents (sEPSCs/sIPSCs), which represent the spontaneous release of excitatory or inhibitory neurotransmitters, primarily glutamate or GABA, from the presynaptic terminals onto postsynaptic neurons [48, 49]. Compared to their WT counterparts, 16p11.2${}^{+/-}$ mice did not show significant changes in either sEPSC amplitude (Additional file 1: Fig. S4A and B) or frequency (Additional file 1: Fig. S4A and C), indicating that frequency of excitatory presynaptic membrane vesicle release and/or the reactivity and density of the excitatory postsynaptic membrane receptors in the hippocampal DG region remained unchanged between the two groups. However, 16p11.2${}^{+/-}$ mice exhibited a significant reduction in sIPSC amplitude (Fig. 3C, D; *p* = 0.002), accompanied by a notable increase in sIPSC frequency, reaching marginal significance (Fig. 3C, E; *p* = 0.042). These patch-clamp results suggest a profound decrease in GABA synthesis or in the amount of GABA translocated into presynaptic membrane vesicles. Additionally, there appears to be a compensatory mechanism involving a faster rate of vesicle release from inhibitory presynaptic membranes. Collectively, these results suggest an unbalanced ratio of excitatory and inhibitory transmitters in the hippocampus of 16p11.2${}^{+/-}$ mice, which may contribute to their social novelty deficits.

Vesicular glutamate transporter 1 (VGLUT1) plays an essential role in regulating glutamate release at the synapse, thereby modulating neuronal excitability. On the other hand, glutamic acid decarboxylase 65 and 67 (GAD65/67) are key enzymes that synthesize GABA, which sustains inhibitory signaling. To confirm the imbalance of excitatory and inhibitory synaptic transmission in the hippocampal DG region of 16p11.2${}^{+/-}$ mice, we assessed the expression of VGLUT1 (Fig. 3F) and GAD65/67 (Fig. 3H), respectively. Our results showed that the expression of VGLUT1 in the hippocampus was unchanged between 16p11.2${}^{+/-}$ and WT mice (Fig. 3G). In contrast, GAD65/67 expression showed a significant decline in the 16p11.2${}^{+/-}$ group (Fig. 3I), indicating disrupted homeostasis of inhibitory and excitatory neurotransmitters in 16p11.2${}^{+/-}$ mice, likely leading to decreased GABA production. Then, we directly assessed the hippocampal glutamate and GABA at the metabolite level using ELISA. As expected, the ELISA results showed an unchanged glutamate level (Fig. 3J) but a significantly decreased GABA level (Fig. 3K) in 16p11.2${}^{+/-}$ mice compared to WT mice. Taken together, our findings suggest that impaired inhibitory transmission in the hippocampus may be an actor responsible for the observed social and behavioral deficiencies observed in 16p11.2${}^{+/-}$ mice.

### IPA reversed defects of social behavior and inhibitory synaptic transmission in DG of 16p11.2${}^{+/-}$ mice

**Fig. 4:**
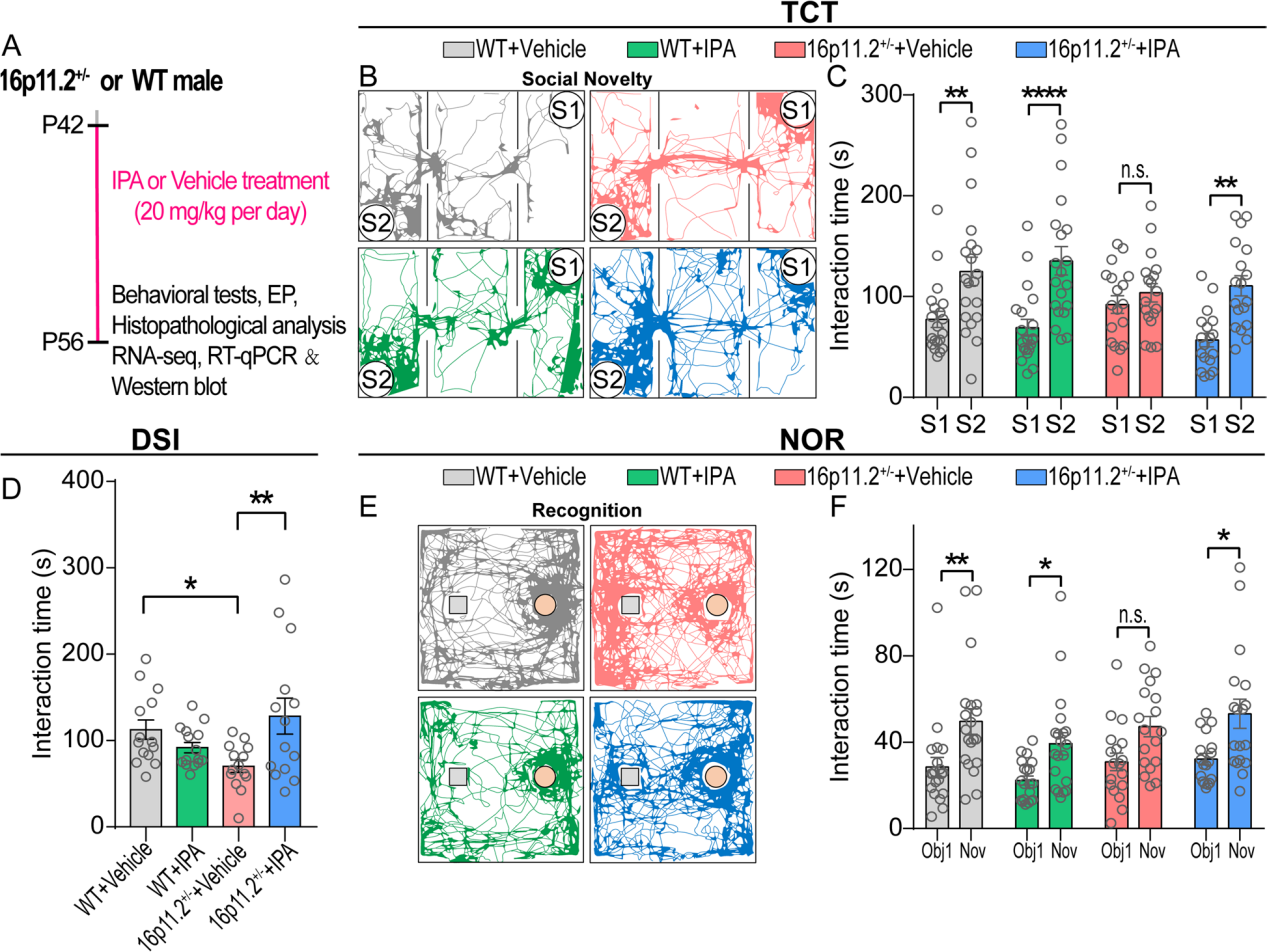
IPA rescued the social novelty deficits and cognitive impairment in 16p11.2${}^{+/-}$ mice. **A** Schematic diagram for experimental design. **B** Representative trajectories of mice in the social novelty phase of the TCT. **C** IPA rescued social novelty deficits of 16p11.2${}^{+/-}$ mice in the TCT (WT + Vehicle: *n* = 20 mice; WT + IPA: *n* = 20 mice; 16p11.2${}^{+/-}$ + Vehicle: *n* = 18 mice; 16p11.2${}^{+/-}$ + IPA: *n* = 18 mice. Two-way ANOVA). **D** IPA improved social interaction time of 16p11.2${}^{+/-}$ mice in the DSI test (WT + Vehicle: *n* = 14 mice; WT + IPA: *n* = 15 mice; 16p11.2${}^{+/-}$ + Vehicle: *n* = 14 mice; 16p11.2${}^{+/-}$ + IPA: *n* = 14 mice. Two-way ANOVA). **E** Representative trajectories of mice in the recognition phase of the NOR test.** F** IPA restored cognitive impairment of 16p11.2${}^{+/-}$ mice but had no significant effect on WT mice in NOR test (WT + Vehicle: *n* = 20 mice; WT + IPA: *n* = 20 mice; 16p11.2${}^{+/-}$ + Vehicle: *n* = 18 mice; 16p11.2${}^{+/-}$ + IPA: *n* = 18 mice, Two-way ANOVA). Data are presented as mean ± SEM. *p < 0.05, **p < 0.01, ****p < 0.0001, and n.s.: not significant. Detailed statistical information is presented in Additional file 2: Table S1

Intestinal metabolite analysis in 16p11.2${}^{+/-}$ mice revealed significant differences in multiple metabolites compared to the WT group. Seeing as IPA was identified as a differential microbial metabolite that is most significantly associated with GM dysbiosis, we hypothesize that IPA may have a positive impact on the autism-associated behavioral defects observed in 16p11.2${}^{+/-}$ mice. To test this hypothesis, we orally gavaged mice with 20 mg/kg IPA or vehicle (i.e., drinking water containing 5% DMSO substituted for IPA) daily for 2 weeks starting from postnatal day 42 (P42), and subjected them to behavioral tests subsequently (Fig. 4A). Strikingly, we found that IPA administration significantly mitigated the social novelty decline in 16p11.2${}^{+/-}$ mice (TCT, Fig. 4B, C and Additional file 1: Fig. S5A). We further observed a significant increase in social interaction time in the DSI test (Fig. 4D) and significant improvement in cognitive ability in the NOR test (Fig. 4E, F and Additional file 1: Fig. S5B) after IPA treatment. Ultrasonic vocalization (USV), produced by various animals, particularly rodents, serves as a crucial means of communication between conspecifics. It also provides an excellent avenue for studying and understanding the social and emotional dimensions of communication in the context of animal behavior and neuroscience [50]. Hence, we further attempted to investigate potential changes in verbal communication in adult male 16p11.2${}^{+/-}$ mice compared to WT controls, and the potential effects of IPA on this behavior. We conducted a USV analysis using a novel three-phase male-female social interaction test. During the first phase of male-female socializing, we observed a significant reduction in the number of vocalizations made by 16p11.2${}^{+/-}$ mice compared to WT mice. Notably, administration of IPA resulted in a significant increase in the number of vocalizations emitted by 16p11.2${}^{+/-}$ mice (Additional file 1: Fig. S5D). Furthermore, we found no significant difference in the total number of calls made by 16p11.2${}^{+/-}$ mice in Phases 2 and 3, compared to WT mice (Additional file 1: Fig. S5E, F). Taken together, our findings indicate that young adult male 16p11.2${}^{+/-}$ mice exhibited significantly reduced vocalization when initially presented to unfamiliar female mice, suggesting a defective response to new social cues.

**Fig. 5:**
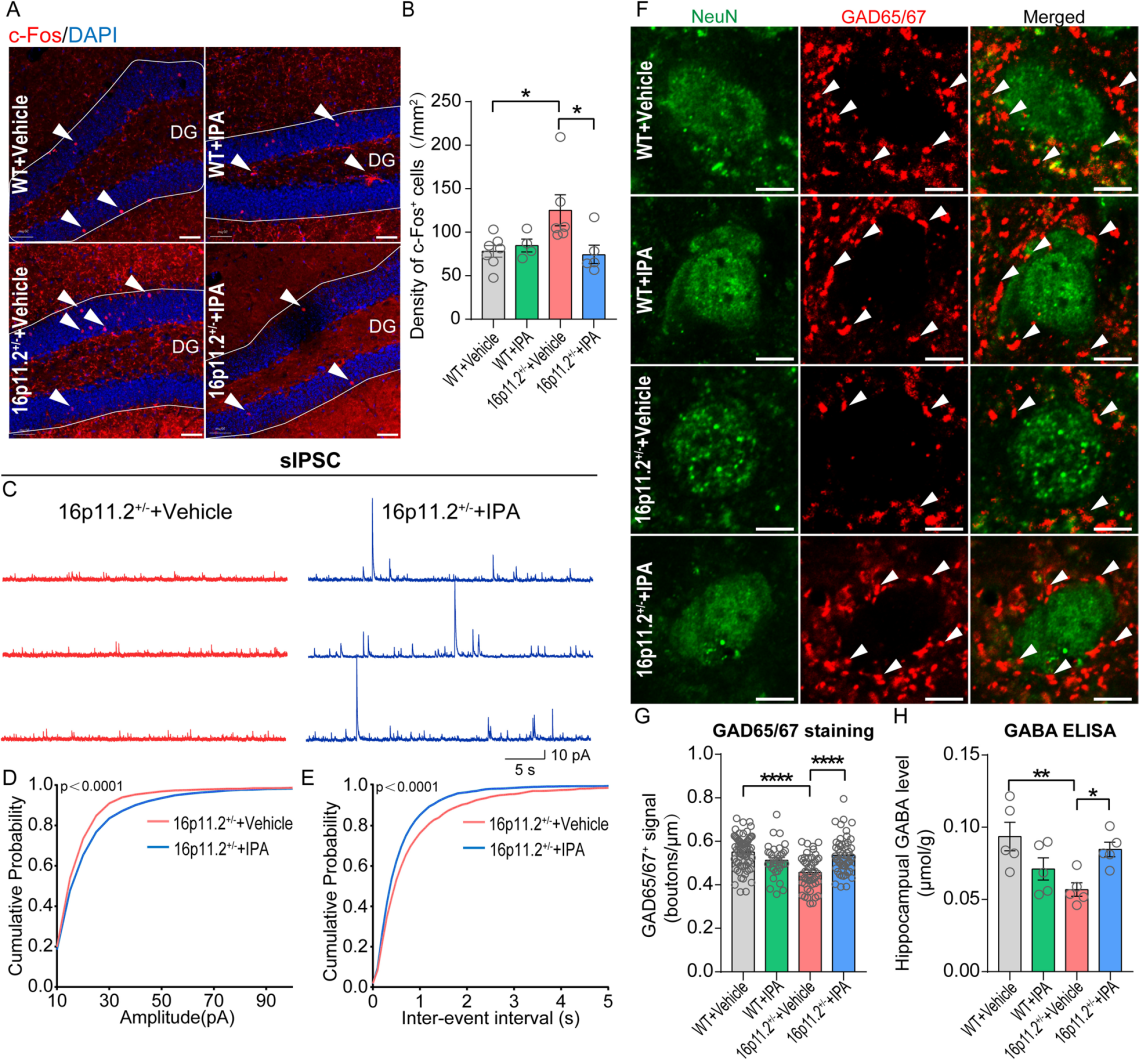
IPA mitigated the imbalance of hippocampal inhibitory transmission in 16p11.2${}^{+/-}$ mice. **A** The red puncta indicate c-Fos^+^ neurons in the DG of the hippocampus. Scale bar: 50 μm. **B** IPA can improve the overactivation of hippocampal neurons in 16p11.2${}^{+/-}$ mice (WT + Vehicle: *n* = 7 mice; WT + IPA: *n* = 4 mice; 16p11.2${}^{+/-}$ + Vehicle: *n* = 6 mice; 16p11.2${}^{+/-}$ + IPA: *n* = 5 mice. Two-way ANOVA). **C** Representative sIPSCs traces from granule cells in hippocampus of mice, scale bars: 5 s, 10pA. **D** Cumulative distribution of sIPSCs amplitudes. **E** Cumulative distribution of sIPSC frequencies (16p11.2${}^{+/-}$ + Vehicle: *n* = 1814 events from 9 cells of 5 mice; 16p11.2${}^{+/-}$ + IPA: *n* = 3169 events from 11 cells of 4 mice. Kolmogorov-Smirnov test). **F** Single confocal planes of punctate GAD65/67 surrounding neurons in the DG of the hippocampus, scale bar: 5 μm. **G** Graph showing the density of GAD65/67 puncta on the perisomatic region of hippocampal neurons (WT + Vehicle: *n* = 68 cells from 5 mice; WT + IPA: *n* = 35 cells from 4 mice; 16p11.2${}^{+/-}$ + Vehicle: *n* = 49 cells from 5 mice; 16p11.2${}^{+/-}$ + IPA: *n* = 52 cells from 5 mice. Two-way ANOVA). **H** IPA restored the level of GABA in hippocampus of 16p11.2${}^{+/-}$ mice (*n* = 5 per group. Two-way ANOVA). Data are presented as mean ± SEM. *p < 0.05, **p < 0.01, and ****p < 0.0001. Detailed statistical information is presented in Additional file 2: Table S1

Immunostaining results demonstrated that IPA substantially normalized the c-Fos hyperactivity pattern in the DG of the hippocampus in 16p11.2${}^{+/-}$ mice (Fig. 5A, B). Furthermore, patch-clamp recordings of brain slices in the hippocampal DG area (Fig. 5C) revealed that IPA significantly increased the amplitude (Fig. 5D) and frequency (Fig. 5E) of sIPSCs in neurons in 16p11.2${}^{+/-}$ mice, which implied that IPA may increase the synthesis of GABA and accelerate the release of inhibitory presynaptic vesicles. In line with these findings, IPA administration significantly increased the expression of GAD65/67 (Fig. 5F, G) and enhanced the level of GABA (Fig. 5H) up to a normal level. Notably, IPA supplementation did not alter the behavioral phenotype, hippocampal neuronal activity, or GABA synthesis in WT mice, as evidenced by comparing relevant readouts between the WT + Vehicle group and the WT + IPA group (Fig. 5B, H). These findings suggest a specific therapeutic potential of IPA on 16p11.2${}^{+/-}$-associated defects in social behavior and the underlying GABA metabolism.

### IPA promoted ERK1/2 phosphorylation in hippocampus of 16p11.2${}^{+/-}$ mice

**Fig. 6:**
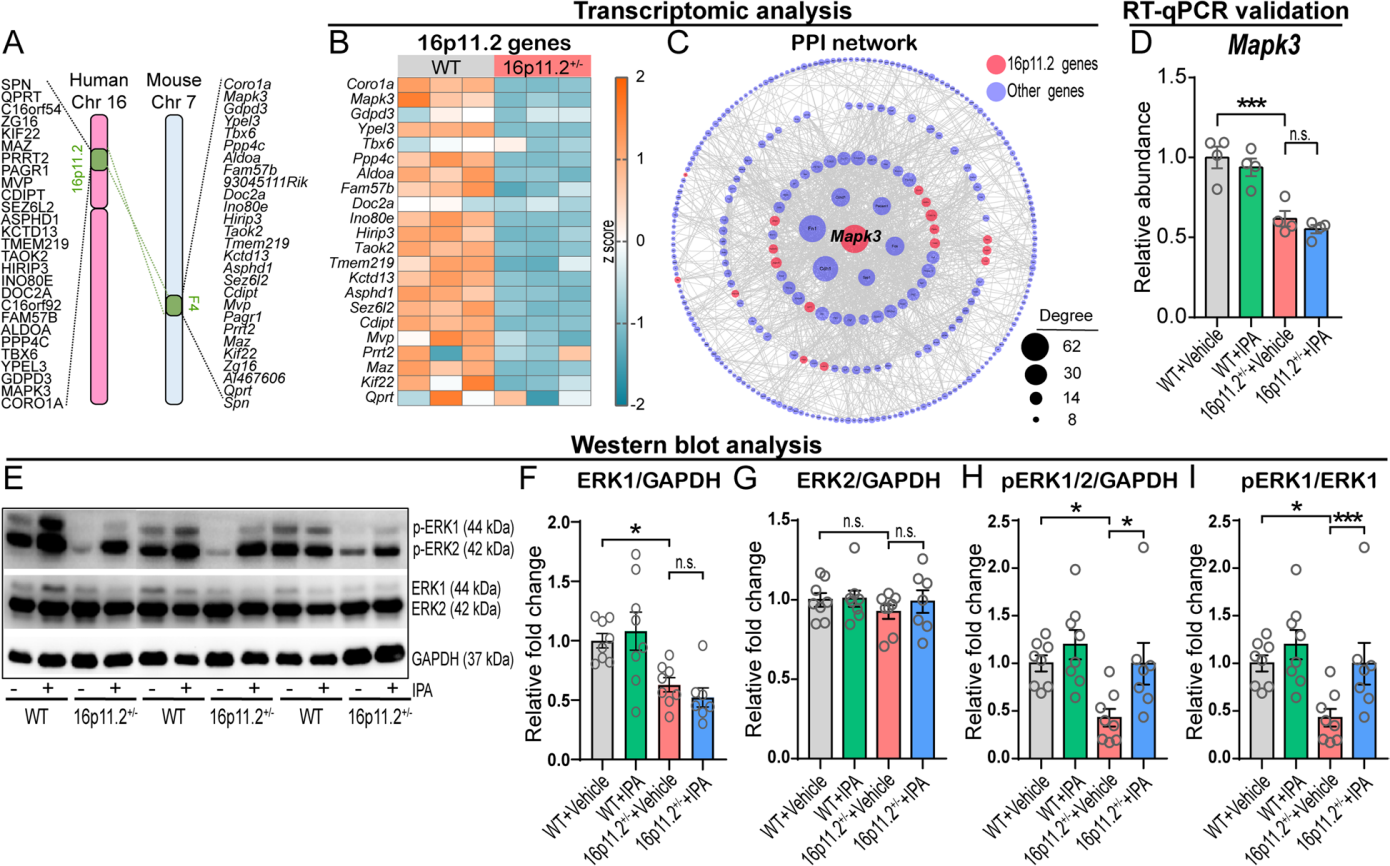
IPA enhanced ERK1/2 phosphorylation in hippocampus of 16p11.2${}^{+/-}$ mice. **A** Schematic of the 16p11.2 deletion region and the synonymous region in mouse chromosome 7. **B** Heat map of 23 expressed genes within 16p11.2 fragment in mouse hippocampus (*n* = 3 per group). **C** Protein-protein interaction (PPI) network of differential genes (screening criteria, *p* < 0.05) in the hippocampus of WT and 16p11.2${}^{+/-}$ mice. Nodes were size-scaled by degree. **D** *Mapk3* expression was decreased in the hippocampus of 16p11.2${}^{+/-}$ mice as assessed by RT-qPCR, and IPA could not increase its expression (*n* = 4 per group. Two-way ANOVA). **E** The representative Western blots showed IPA promoted the phosphorylation of ERK1/2 in hippocampus of 16p11.2${}^{+/-}$ mice. **F**-**I** Quantification of Western blot analysis showed that IPA did not change the expression levels of ERK1 (**F**) and ERK2 (**G**) in the hippocampus of 16p11.2${}^{+/-}$ mice, but significantly increased the phosphorylation level of ERK1/2 (**H**, **I**) (WT + Vehicle: *n* = 8 mice; WT + IPA: *n* = 8 mice; 16p11.2${}^{+/-}$ + Vehicle: *n* = 8 mice; 16p11.2${}^{+/-}$ + IPA: *n* = 7 mice. Two-way ANOVA). Data are presented as mean ± SEM. *p < 0.05, **p < 0.01, ***p < 0.001, and n.s.: not significant. Detailed statistical information is presented in Additional file 2: Table S1

Given our findings that loss of 16p11.2-associated genes elicited GM dysbiosis, social behavior defects, and perturbed hippocampal synaptic inhibition that were substantially rescued by IPA supplementation, we sought to understand the potential impact of IPA on the expression and function of specific ASD-associated genes within the 16p11.2 region (Fig. 6A). In so doing, we first screened for the key 16p11.2 genes that contribute to hippocampal dysfunction using RNA sequencing on hippocampal tissue from 16p11.2${}^{+/-}$ and WT mice. Among 27 coding genes in the 16p11.2 locus, we detected 23 in the hippocampus, and 19 of these, including *Mapk3*, were significantly reduced in the 16p11.2${}^{+/-}$ group (Fig. 6B).

As previous studies have implicated single genes within the 16p11.2 locus in neurodevelopmental diseases and ASD [7–9], we aimed to identify hub genes with important biological significance in the disease pathway and potential therapeutic targets [51]. Using the STRING database, we assessed protein-protein interactions (PPI) of the full repertoire of 524 significantly differentially expressed genes (DEGs, *p* < 0.05) in the hippocampus of 16p11.2${}^{+/-}$ mice relative to WT peers (Additional file 3: Table S2), and found that 293 genes formed a PPI network. Strikingly, *Mapk3* showed the highest betweenness centrality (BC = 0.163), closeness centrality (CC = 0.485), and degree of connectivity (D = 62) among those PPI network genes, indicating that *Mapk3*/ERK1 may serve as the most central topological node in the PPI network (Fig. 6C and Additional file 4: Table S3).

To investigate the potential effects of IPA on hippocampal *Mapk3* gene expression, we conducted RT-qPCR analysis to compare total *Mapk3* mRNA levels in the presence and absence of IPA treatment. Surprisingly, we found that IPA did not promote hippocampal *Mapk3* gene expression at the transcription level in either 16p11.2${}^{+/-}$ or WT mice, despite a marked decrease in 16p11.2${}^{+/-}$ mice in the absence of IPA supplementation (Fig. 6D) that is in keeping with our RNA-sequencing result. To further elucidate the potential mechanisms underlying these observations, we investigated whether IPA might affect *Mapk3* expression at the translational and/or post-translational level. Specifically, we examined the expression and phosphorylation levels of ERK1, the protein encoded by *Mapk3* gene. Consistent with our RT-qPCR results, we observed a significant decrease in ERK1 expression in the hippocampus of 16p11.2${}^{+/-}$ mice, while IPA had no significant effect on ERK1 expression (Fig. 6E, F). Additionally, compared to WT and non-treatment controls, neither 16p11.2${}^{+/-}$ nor IPA had a significant effect on ERK2 expression (Fig. 6G), which is encoded by the *MAPK1* gene on chromosome 22 in humans and shares an array of downstream targets with ERK1, participating in a plethora of cellular processes such as cell proliferation, differentiation, survival, and migration [52, 53]. Intriguingly, we found that IPA significantly increased the phosphorylation of ERK1/2, the activated forms of ERK1/2, in the hippocampus of 16p11.2${}^{+/-}$ mice (Fig. 6H and I). These findings suggest that IPA may play a crucial role in improving behavioral deficits and inhibitory synaptic transmission impairment in 16p11.2${}^{+/-}$ mice by enhancing the functional activity of ERK.

## Discussion

Emerging evidence, particularly from clinical studies on ASD, suggests that microbiome plays a pivotal role in modulating behavioral symptoms and brain function in individuals with ASD [30, 32]. In addition, alterations in GM and metabolites have been observed in multiple mouse models of ASD, such as the *Shank3B*^−/−^, *Chd8*${}^{+/-}$, and BTBR mouse models [14, 21, 54]. Nevertheless, the complete impact of specific metabolites in the gut microbiota on brain function, cognition, and social behavior has yet to be fully elucidated.

In this study, we investigated the relationship between the microbiota-metabolites-brain axis and the mechanisms of action of key metabolism in 16p11.2${}^{+/-}$ mice. By profiling the gut microbial components and metabolism, we revealed that 16p11.2${}^{+/-}$ mice had a disrupted intestinal flora characterized by a significant reduction in the levels of IPA (Fig. 1). Further, we found that social deficits and cognitive memory impairment in 16p11.2${}^{+/-}$ mice were significantly ameliorated after chronic IPA supplementation (Figs. 2 and 4). Strikingly, IPA supplementation could reverse abnormal neuronal activation and mitigate impaired inhibitory synaptic transmission in the DG area of the 16p11.2${}^{+/-}$ hippocampus (Figs. 3 and 5). Subsequently, we investigated the underlying mechanism of IPA action and found that IPA administration significantly increased ERK1/2 phosphorylation in the 16p11.2${}^{+/-}$ hippocampus, while ERK1 (encoded by the *MAPK3* gene within the 16p11.2 region) remained unchanged at the transcriptional and translational level (Fig. 6).

Recent research has identified several small molecule compounds derived from the gastrointestinal tract that play key roles in neuropsychiatric disorders, such as ASD and anxiety [55, 56]. Among these compounds, the levels of IPA may change during the progression of different diseases. For example, patients with chronic kidney disease exhibit significantly lower serum IPA levels [57], and whole-body or abdominal irradiation in mice leads to a significant decrease in fecal IPA levels [35]. IPA is produced through intestinal flora by tryptophan metabolism and plays a neuroprotective role in the central and peripheral nervous systems, further supporting its role as a mediator in the gut-brain axis connection [25, 28, 58]. Interestingly, our study found that both fecal and serum IPA content was significantly reduced in 16p11.2${}^{+/-}$ mice, along with a reduction in the richness of *C. sporogenes*, the primary producer of IPA in feces. Furthermore, chronic treatment with IPA significantly alleviated the social deficits and memory impairment observed in 16p11.2${}^{+/-}$ mice, highlighting it as a vital mediator for such abnormal behavior.

In addition to social impairments, individuals with 16p11.2${}^{+/-}$ exhibit deficits in various cognitive domains, including memory, verbal and nonverbal IQ, and cognitive flexibility [11, 59]. Importantly, impaired memory can also have a significant impact on academic performance, social interaction, and adaptive functioning [60, 61]. Moreover, the cognitive and memory abilities in animals often serve as important indicators when detecting autism-related behaviors in rodents [40, 62]. Consequently, we assessed the cognitive memory abilities of 16p11.2${}^{+/-}$ mice through NOR experiments. Indeed, our study confirms that 16p11.2${}^{+/-}$ mice show memory deficits in tasks such as the TCT and the NOR experiments, in keeping with those reported previously [40, 63, 64]. While the direct impact of IPA on cognitive memory deficits stemming from neurodevelopmental anomalies in either humans or animals is relatively constrained, IPA, functioning as a potent neuroprotective agent, has already exhibited its capacity to ameliorate spatial memory impairments induced by cecal ligation puncture [65]. Notably, a recent investigation has unveiled IPA’s capability to enhance spatial memory and alleviate social impairments in rat models subjected to prenatal caffeine exposure (PCE)-induced intrauterine growth restriction (IUGR) [66]. In addition, IPA holds promise as a therapeutic intervention for Alzheimer’s disease, a condition characterized by profound memory loss [26, 67]. Our research outcomes, along with those from prior studies, underscore the prospective efficacy of IPA in ameliorating both memory and social interaction deficits.

The intricate processes of memory encoding, consolidation, and retrieval involve distinct roles played by various subregions of the hippocampus, such as the DG, CA1, and CA3, yet the precise nature of their contributions remains enigmatic [68]. The hippocampal DG region holds particular significance in the regulation of social behavior [45]. Numerous studies have highlighted the crucial role of the DG in the context of social behavior deficits with ASD. For instance, research by Mohammadkhani et al. demonstrated that prenatal exposure to valproic acid induces synaptic plasticity defects in the DG region, potentially contributing to social interaction deficits in rats with ASD [69]. Additionally, Cai et al. reported that the abnormal development of the DG region in mice’s hippocampus is associated with deficits in social interaction [70]. Furthermore, the DG stands out as one of the few brain regions where adult neurogenesis occurs. The reduction in neurogenesis within the DG region in a mouse model of ASD has been identified to contribute to deficits in social behavior [71]. Taken together, our focus on the DG region as the primary area of interest is grounded in its relevance to abnormal behavior in the observed mouse models, its role in synaptic plasticity, and its involvement in adult neurogenesis, all of which are considered critical aspects in understanding the neural basis of social behavior deficits.

Investigations involving 16p11.2${}^{+/-}$ rat models revealed unchanged counts and distributions of excitatory and inhibitory neurons within the CA1 region, albeit an observed hyperexcitation of somatostatin-type interneurons in CA1 [72]. However, research on 16p11.2${}^{+/-}$ mice has uncovered heightened excitability, an imbalance between excitatory and inhibitory functions, and accelerated glutamatergic synapse maturation within hippocampal CA1 neurons during early postnatal development [73]. Further, alterations in metabolic glutamate receptor 5 (mGluR5)-dependent synaptic plasticity and protein synthesis within the CA1 hippocampal region have been identified as contributing factors influencing the memory functionality of these animals [64]. While previous evidence has indicated intact long-term potentiation and long-term inhibition of synaptic transmission in hippocampal CA1 region of 16p11.2${}^{+/-}$ mice [63, 64], our findings draw attention to the synaptic transmission function in the hippocampal DG region.

In our study, despite a discernible trend in the CA1 region, abnormal neuronal activation was particularly evident in the DG region, potentially implicating its role in affecting memory function. Subsequent electrophysiological assessments showed anomalies in postsynaptic membrane spontaneous discharge in the DG region, characterized by a significant decrease in sIPSC amplitude and an increase in frequency. These results suggest disturbances in synaptic transmission within the hippocampal DG region, potentially contributing to the cognitive memory and social impairment in 16p11.2${}^{+/-}$ mice. In keeping, the modulation of synaptic plasticity in DG neurons in rats is intricately associated with key processes, including social interaction and the formation of memory [69]. Despite no significant changes in dendritic branching and synaptic density of granule cells in the DG region (Additional file 1: Fig. S6 and S7), we observed an imbalance in inhibitory transmitter delivery. Taken together, these distinct synaptic phenotypes in subregions of the hippocampus of 16p11.2${}^{+/-}$ mice, featuring an imbalance of neurotransmitters, indicate potential impact of regional or cell-type-specific alterations on social and memory deficits [10].

The hypothesis of E-I imbalance in the brain has been proposed as a potential pathogenesis of ASD [1]. Abnormal neurotransmitter synthesis or transformation, such as metabolic conversion between glutamate and GABA, could result in brain function disorders. Several studies have found that the concentration of GABA is significantly decreased in children with ASD in various brain regions, including the visual and auditory areas [74, 75]. Mouse models with 16p11.2 CNV exhibit synaptic dysfunction and E-I imbalance in the somatosensory cortex, the mPFC, and the hippocampus [23, 64, 76]. In our study, we observed significantly reduced expression of GAD65/67 and levels of GABA in the hippocampus of 16p11.2${}^{+/-}$ mice, and the impaired inhibitory synaptic transmission was restored by IPA through the normalization of GABA levels. Notably, following the TCT, neurons in the DG region exhibited heightened activation, as evidenced by a marked increase in c-Fos^+^ cells. Elevated c-Fos expression often indicates intensified neuronal activation [77]. Utilizing immunofluorescence staining, we found that the activated cells were predominantly situated within the granule cell layer (Fig. 5G, H), implying that a diminished presence of inhibitory transmitters, such as GABA, may render granule cells more susceptible to activation, thereby contributing to the observed increase in c-Fos expression. Hence, such heightened activity may arise either from a reduction in inhibitory input onto these neurons, as delineated by our data, or from an augmentation of excitatory input, a facet that warrants future investigation. Nonetheless, a decline in GABAergic signaling, as indicated in our study, can precipitate a state of disinhibition, weakening inhibitory control over specific neurons [78]. Consequently, our findings propose a dual effect: a decrease in GABA content in the hippocampus coupled with an augmentation of granule cell activity in the DG region. This interplay may contribute to the observed aberrant neuronal activation and altered GABAergic signaling in 16p11.2${}^{+/-}$ mice following the TCT. Therefore, targeting the glutamate and GABA system to restore E-I balance and synaptic plasticity is considered a promising intervention approach for 16p11.2 CNV syndrome [10].

Typically, hippocampal DG receives input from the entorhinal cortex through granule cells, which project their axons, known as mossy fibers, to the CA3 region. Subsequently, CA3 neurons project to CA1 via the Schaffer collateral pathway, completing the information transmission [79, 80]. Of note, while CA1 serves as a hub for integrating information from DG and CA3, CA1 neurons could also directly receive input from the entorhinal cortex [81]. In our study, we observed abnormal neuronal activation in the hippocampal DG region following the TCT. While CA1 neurons also exhibited a trend of increased activation (*p* = 0.058), no apparent abnormalities were observed in the CA3 region. This activation pattern appears to deviate from the anticipated DG-CA3-CA1 activation sequence. It is crucial to recognize that distinct subregions of the hippocampus serve specific functions. For example, CA1 is essential for memory consolidation, spatial navigation, and retrieval, while CA3 plays a key role in pattern completion, associative memory, and the rapid encoding of information [82, 83]. Activation patterns in DG, CA3, and CA1 can vary depending on the task, stimulus, or memory process involved. Coordinated activation may span the entire DG-CA3-CA1 network in some instances, while in others, activation may be more selective based on specific cognitive and/or social demands [83, 84]. Therefore, the non-sequential activation in 16p11.2${}^{+/-}$ mice is intriguing and warrants further research to elucidate the underlying circuitry mechanisms.

Electrophysiologically, GABA activates the α1, α2, and γ2 subunits of the GABA_A_ receptors to generate IPSCs [85]. The amplitude of IPSCs in the hippocampus is determined by the levels of GABA and GABA_A_ receptors [86, 87]. Overexcitation of neural networks due to reduced inhibitory neurotransmitters is a phenomenon frequently observed in individuals with ASD [88, 89]. Our electrophysiological recordings reveal a significant decrease in the amplitude of sIPSC (*p* < 0.001) in granule neurons of 16p11.2${}^{+/-}$ mice, concomitant with an increase in sIPSC frequency (*p* = 0.042). Of note, there is a statistically significant disparity between the sIPSC amplitude and frequency. Subsequent findings on the diminished levels of GABA and GAD65/67 within the hippocampal DG region of 16p11.2${}^{+/-}$ mice (Fig. 5G, H) confirm a substantial decrease in inhibitory transmitter availability to granule cells. In keeping, the aberrant activation observed in the hippocampal DG neurons supports a plausible explanation for the diminished GABA levels within this region. These findings collectively suggest that, owing to the reduced GABA availability, a profound decline in sIPSC amplitude primarily contributes to the heightened excitability of the neural network. Concurrently, the observed increase in sIPSC frequency may act as a compensatory and counteracting response to mitigate the effects of aberrant neuronal activation. This nuanced interplay between reduced inhibitory strength and compensatory changes in frequency provides insights into the intricate mechanisms underlying the observed alterations in synaptic transmission and network excitability in 16p11.2${}^{+/-}$ mice. Furthermore, administration of IPA significantly increased the amplitude of sIPSC in DG neurons, supporting our conclusion that IPA promotes GABA synthesis or transport in the hippocampus of 16p11.2${}^{+/-}$ mice.

The formation and elimination of synapses is crucial to the proper assembly of neural networks and the maintenance of nervous system function [90]. This significance is particularly pronounced in neuropsychiatric disorders such as ASD, where alterations in synaptic pruning, plasticity, and dysregulation of synaptic circuits are pivotal contributors to the manifestation and severity of ASD symptoms [91], often coinciding with critical stages of synaptic pruning. Furthermore, the impact of dysregulated gut flora on neuronal circuit function through altered synaptic pruning is equally significant [92]. In our investigation of the microbiota composition in 16p11.2${}^{+/-}$ mice, notably, we observed an imbalance, particularly the significant reduction of the gut microbial metabolite IPA, which was associated with abnormal synaptic transmission of neurons. Wang et al. reported the role of IPA in mitigating ASD-like behavior in a rat model of IUGR, where IPA effectively regulates the overactivation of hippocampal microglia and prevents excessive pruning of neuronal synapses by modulating the AHR/NF-κB signaling pathway [66]. Concurrently, our research, conducted using 16p11.2${}^{+/-}$ mice, yielded intriguing results as we delved into the dendritic branching and synaptic density of granule neurons within the DG region of 16p11.2${}^{+/-}$ mice and the potential impact of IPA on synaptic pruning. Our subsequent biocytin infusion of granule neurons and 3D reconstruction of the dendritic branching revealed no significant alterations in the complexity of dendritic branches or the density of dendritic spines of the granule cells between 16p11.2${}^{+/-}$ and WT mice. Further, the administration of IPA did not significantly affect these parameters in either 16p11.2${}^{+/-}$ or WT mice (Additional file 1: Fig. S6). Similarly, our examination of the density of neuronal dendritic spines via Golgi staining showed no significant differences in 16p11.2${}^{+/-}$ versus WT mice (Additional file 1: Fig. S7). Collectively, our results suggest a potential dominant role of the identified imbalance of inhibitory transmitter delivery in contributing to the observed behavioral deficits in the 16p11.2${}^{+/-}$ mice, where dendritic branching and synaptic density of granule cells in the DG region remain unchanged.

The preponderance of evidence reported in the present study supports the conclusion that IPA has great potential as a novel therapeutic target for 16p11.2 deletion syndrome, as well as for other disorders characterized by cognitive and social behavior defects. Notably, some indole derivatives, such as serotonin (5-HT) and melatonin, which have similar chemical structures to IPA, play important roles in regulating physiological processes in the body [93]. For instance, melatonin can rapidly enhance GABA-induced current generation in rat hippocampal neurons and increase the amplitude and frequency of miniature IPSCs current, indicating its effect on the enhancement of the GABAergic system [94]. Additionally, both 5-HT supplementation and activation of 5-HT type 2C receptors can increase GABA release [95, 96]. Collectively, these findings suggest that the modulation of social behavior in 16p11.2${}^{+/-}$ mice by IPA is likely due to its role in regulating inhibitory transmitters in the neurons of the hippocampus.

The molecular mechanism underlying the function of IPA in the CNS remains poorly understood. Emerging evidence suggests that the activity of the ERK signaling in the 16p11.2${}^{+/-}$ mice varies in different brain regions and developmental stages. For instance, phosphorylation of ERK1/2 was increased at the embryonic stage (E14.5) and postnatal day 10 (P10) in the cortex of 16p11.2${}^{+/-}$ mice [97, 98]. Our study and a previous one [64] found that the levels of ERK1 and phospho-ERK1 were decreased in the hippocampus of adult 16p11.2${}^{+/-}$ mice. Of particular interest, our findings demonstrated that IPA treatment rescued the reduction of ERK1/2 phosphorylation level, without altering the expression of ERK1 and its coding gene, *Mapk3*. To the best of our knowledge, the regulatory effect of IPA on the ERK pathway has not been reported before. A series of behavioral experiments have confirmed that ERK is involved in the process of learning and memory, and notably, the activation of ERK in hippocampus can effectively enhance the ability of learning and memory [99–101]. However, the ERK signaling pathway is complex and interacts with other molecular network, engaging a myriad of cytoplasmic and nuclear substrates that regulate multiple cellular processes. Therefore, disruption of homeostasis in ERK signaling may result in cognitive memory and behavioral impairment [102]. Nonetheless, further studies are required to determine precisely how IPA promotes ERK phosphorylation. Overall, our study suggest that IPA supplementation can substantially improve hippocampal inhibitory synaptic transmission impairment and behavioral deficits in 16p11.2${}^{+/-}$ mice, possibly through the activation of ERK signaling pathway.

## Conclusion

Our study demonstrates that IPA, a metabolite produced by intestinal microbiota from tryptophan, has a significant impact on mitigating behavioral deficits and imbalanced inhibitory synaptic transmission in the hippocampus of mice with a 16p11.2 copy number variation. Furthermore, our results showed that IPA significantly enhances the phosphorylation level of ERK1, a protein encoded by the *MAPK3* gene in the 16p11.2 locus. Overall, our findings reveal a novel role of IPA in improving social memory and inhibitory neurotransmission and provide new insights into its potential applications for interventions aimed at addressing behavioral deficits associated with 16p11.2 syndrome.

## Methods

### Mice

16p11.2${}^{+/-}$ mice were obtained from Jackson Laboratories (Stock No: 013128) and maintained on a C57BL/6 J background. To avoid possible behavioral alterations in maternal care resulting from genetic mutants [103], we mated male 16p11.2${}^{+/-}$ mice with female WT mice for breeding (Fig. 1A). All mice used in our research were male. Male 16p11.2${}^{+/-}$ and WT littermates were separated at 3 weeks old and were raised until 8 weeks old for behavioral tests, electrophysiological analysis, and histopathological analysis (Fig. 1B). All mice were housed in controlled, appropriate specific pathogen-free conditions with a constant temperature at 22 ± 1 °C and humidity at 50%, and separated after weaning according to their genotype and sex. The animal room was kept on a 12 h light/dark cycle (light on at 7 a.m.). All experimental procedures were approved by the Animal Care and Use Committee of Southern University of Science and Technology, China.

### Behavioral tests

Mice were handled for 3 days before the behavioral tests and transferred in the test room for at least 30 min before each experiment. All tests were conducted between 9:00 a.m. and 7:00 p.m. Eight-week-old male mice were used for standardized behavioral tests.

### Three-chamber test

The TCT was performed as previously described [54]. Firstly, mice were placed in an empty three-chamber apparatus (60 × 40 × 20 cm, L × W × H) that was divided into three interconnected chambers for 10 min. In a second 10-min period, the test mice could interact either with a small empty cage (E) placed in the left chamber, or with a genotype-, age-, and sex-matched stranger mouse (S1) placed in a cage in the right chamber. Subsequently, in the third phase, we examined the mice’s social novelty preference. Briefly, we placed a novel stranger mouse (S2) in the previously empty cage, and then recorded the contact time of the test mice with the S1 or S2 mice. Time spent in sniffing the empty cage or S1 or S2 mice was recorded and measured using the Noldus EthoVision XT 10 software (Noldus Information Technology, USA).

### Direct social interaction test

The DSI test was performed in a neutral cage (gray Plexiglas box, 30 × 20 × 20 cm). The social interaction time between the test mice and unfamiliar mice that matched according to age and sex was recorded. The social interaction behaviors of mice mainly included touching, nose-to-nose sniffing, nose-to-anus sniffing, close following, and crawling over/under each other.

### Novel object recognition

The NOR test was adapted from experiments previously described [40]. Mice were put into a box (40 × 40 × 40 cm, L × W × H) to acclimatize for 10 min and then returned to their home cage. Twenty-four hours after habituation, the mice were exposed to two identical objects (Obj1, Obj2) for 10 min. Two hours after object exploration, one object was replaced with a novel object (Nov) and the mice were allowed to explore the Obj1 and Nov for 10 min. Time spent sniffing within 2 cm of each object or directly touching the objects was recorded.

### Open field test

The OFT was performed as previously described [104]. The mice were introduced to an open box (40 × 40 × 40 cm, L × W × H) and allowed to explore for 10 min. Total distance traveled, time spent in the center (20 × 20 cm), and movement speed were recorded by Noldus EthoVision XT software.

### Elevated Plus-Maze

EPM was formed by two closed and open arms, standing 1 m above the floor. The mice were placed in the center area, facing an open arm, and allowed to explore freely for 5 min. The number and duration of mouse access to each area were recorded with a video tracking system and automatically measured by Noldus EthoVision XT10 software. As previously described [105], the anxiety index was calculated as 1 − [(time spent in open arms/total time)/2 + (number of entries to the open arms/total entries)/2].

### Ultrasonic vocalization

The USV analysis was conducted using a novel three-phase male-female social interaction test, as previous described [38, 106]. In brief, a young adult male subject (16p11.2${}^{+/-}$ or WT mice, approximately 8 weeks of age) initially engages in a 5-min interaction with an unfamiliar young adult female mouse of similar age (Phase 1), then spends 3 min alone in the social cage after the female is removed (Phase 2), and finally, re-engages in a 3-min interaction with the previously familiarized female upon her return to the social cage. The number of vocalizations made by male mice was recorded and assessed during each of the three phases.

### Fecal sample collection

Fresh fecal pellets were collected from 8-week-old 16p11.2${}^{+/-}$ or WT mice and stored at −80 °C immediately until microbiota and metabolome analysis.

### DNA extraction and 16S rRNA gene sequencing

Bacterial DNA was extracted from 0.2 g of each fecal sample using the CTAB/SDS method, as previously described [107] and stored at −80 °C for further analysis. The concentration and purity of DNA were assessed on 1% agarose gels. The 16S rRNA V4 region was amplified. Genomic DNA was sequenced on an Illumina NovaSeq platform (Illumina, USA) by Novogene company in Beijing, generating 250 bp paired-end reads. Paired-end reads were merged using FLASH (Version 1.2.11, http://ccb.jhu.edu/software/FLASH/), and the spliced sequences were refferred to as raw tags. Effective tags were obtained from the raw data after filtering to remove the chimeric sequences. Amplicon Sequence Variants (ASVs) denoising and species annotation were performed using QIIME2 software (Version QIIME2-202006). The alpha diversity and beta diversity were calculated in QIIME2. Shannon index and Chao index were analyzed with GraphPad Prism 8.0.

### Fecal metabolomic analysis

The metabolites in stool samples were isolated and detected by vanquish ultra-high-performance liquid chromatograph (UHPLC) coupled with quadrupole-time-of-flight mass spectrometry (Q-TOF MS) system. Briefly, 800μL methanol acetonitrile solution (1:1, v/v) was added to 80 mg of fecal sample, vortexed and centrifuged. The samples were then incubated on ice for 10 min and centrifuged at 14,000 rpm at 4 °C for 20 min, and the supernatant was retained. The supernatant was stored at −80 °C before UHPLC-Q-TOF MS analysis. We used an Agilent 1290 Infinity LC system (Agilent, USA, chromatographic column:1.7 µm, 2.1 mm × 100 mm) to perform chromatographic separation of the samples at a constant temperature of 25 °C and an AB Triple TOF 5600/6600 series mass spectrometer (AB SCIEX, USA) to detect eluted metabolites. Raw data were pretreated using ProteoWizard (http://www.proteowizard.org/) and then peak aligned and quantified for each metabolite using the XCMS program (https://xcmsonline.scripps.edu). The normalized data were subjected to orthogonal partial least squares discriminant analysis (OPLS-DA) and univariate statistics to obtain differential metabolites.

### Bacterial quantification by quantitative PCR (qPCR)

DNA was isolated from stool sample using MolPure Stool DNA Kit (Yeasen Biotechnology, China), according to the manufacturer’s protocol. The total DNA concentration of fecal was determined by NanoDrop. qPCR was performed using a SYBR Green Master Mix (Accurate Biology, China), according to the manufacturer’s protocol. The gene-specific primers of main producer *C. sporogenes* and total bacteria are listed in Additional file 5: Table S4. The primers of the IPA producing-associated genes are listed in Additional file 6: Table S5.

### Drug administration

IPA (Sigma-Aldrich, USA) was orally gavaged at a dose of 20 mg/kg, as previously reported [28]. Briefly, IPA was dissolved in 5% dimethyl sulfoxide (DMSO, MP Biomedicals, USA) in drinking water, and 5% DMSO in drinking water was used as the vehicle control.

### RNA sequencing and PPI analysis

Total RNA was extracted from the hippocampus of adult mice (8 weeks). RNA integrity was assessed using the RNA Nano 6000 Assay Kit of the Bioanalyzer 2100 system (Agilent Technologies, USA). The mRNA was purified from total RNA using poly-T oligo-attached magnetic beads and then reverse transcribed into cDNA. The 370 ~ 420 bp length cDNA fragments were preferentially purified by AMPure XP system (Beckman Coulter, USA). At last, PCR products were purified (AMPure XP system) and library quality was assessed on the Agilent Bioanalyzer 2100 system. Differential expression analysis between WT and 16p11.2${}^{+/-}$ groups was performed using the DESeq2 R package (1.20.0). Genes with a *p*-value < 0.05 identified by DESeq2 were designated as differentially expressed. PPI analysis of differentially expressed genes was performed utilizing the STRING database (https://cn.string-db.org/), which known and predicted protein-protein interactions. Values of degree, betweenness centrality, and closeness centrality were extracted using Cytoscape’s integrated network analysis module (https://github.com/cytoscape/).

### Real-time PCR analysis

The reverse transcription quantitative PCR (RT-qPCR) was performed, as described previously [108]. Firstly, total RNA was extracted from hippocampus using Trizol reagent (Invitrogen, USA). Then, RNA was reversed-transcribed with a cDNA reverse transcription kit (Accurate Biology, China). Real-time PCR was conducted with SYBR Green Master Mix (Accurate Biology, China) using a CFX 96 Real-Time PCR System Detector (Bio-Rad Laboratories, USA). The expression levels of the *Mapk3* gene was normalized against internal control *Gapdh* gene. The following primer sets were used: *Gapdh*, Forward: 5′ -TGTGTCCGTCGTGGATCTGA-3′; Reverse: 5′-CCTGCTTCACCACCTTCTTGA-3′. *Mapk3*, Forward: 5′-CAATGACCACATCTGCTACTTCCTCTAC-3′; Reverse: 5′-TTAAGGTCGCAGGTGGTGTTGATAAG-3′.

### Western blot analysis

After mice were anesthetized with isoflurane (RWD Life Science, China), the hippocampus was quickly removed by using a mouse brain mold and put into liquid nitrogen. The hippocampus was homogenized in RIPA Buffer (Beyotime, China) comprising protease inhibitor (MedChemExpress, USA) and phosphatase inhibitor cocktail (MedChemExpress, USA). The homogenates were centrifuged at 12,000 rpm, 4 °C for 15 min, and the liquid supernatant was collected. The prepared protein solution was loaded onto an SDS-PAGE gel (Yeasen Biotech, China) and transferred to PVDF membranes (Merck Millipore, USA). The PVDF membranes were blocked with 5% fat-free milk (Biofroxx, Germany) in 1× TBST, followed by incubation with primary antibodies at 4 °C overnight. The primary antibodies used were mouse anti-ERK (Millipore, USA, 1:1000 dilution), rabbit anti-p-ERK (Millipore, USA, 1:1000 dilution), and rabbit anti-GAPDH (Affinify, China, 1:3000 dilution). The blotted membranes were washed with TBST and then incubated with HRP-conjugated secondary antibodies (Affinify, China, 1:3000 dilution) at room temperature (RT) for 2 h. Protein immunoreactivity was detected with a Super ECL Detection Kit (Yeasen Biotech, China), and the signals were visualized with the ChemiDocTM Touch imaging system (Bio-Rad Laboratories, USA).

### Enzyme-linked immunosorbent assay (ELISA)

After mice were anesthetized with isoflurane, blood was gained from their eyeballs and centrifuged at 3000 rpm for 10 min. The serum was transferred to a new centrifuge tube and stored frozen for further analysis. The hippocampus of mice was lysed with PBS and homogenized on ice. The supernatant was collected after centrifugation at a speed of 12,000 rpm for 15 min at 4 °C. Both the serum and the supernatant of hippocampus were detected by ELISA kits for IPA (Jianglaibio, China), GABA (Yuanjubio, China), and VGLUT1 (Yuanjubio, China), according to the manufacturer’s instructions.

### Immunofluorescence

Immunofluorescence was performed as we previously described [108]. Briefly, anesthetized mice were transcardially perfused with 20 mL PBS followed by 20 mL 4% paraformaldehyde (PFA) (Macklin, China) immediately. Brain tissues were removed, post-fixed in 4% PFA for 2 days at 4 °C and cryoprotected in 30% sucrose over 2 days. Coronal brain sections were cut at 30 µm thickness with a cryostat (Leica Biosystem, Germany) and then blocked with 10% goat serum (Boster Biological Technology, China) and 0.3% Triton X-100 (Sangon Biotech, China) in PBS at RT for 1 h. Sections were then incubated at 4 °C overnight with primary antibodies diluted in blocking buffer. Sections were washed three times with PBST (0.3% Triton X-100 in 0.1 M PBS) for 10 min each, and incubated in second antibodies at RT for 2 h in the dark. Slices were mounted onto slides and coverslipped with a Fluoroshield Mounting Medium (Beyotime, China). Fluorescence imaging and data acquisition were performed on a ZEISS LSM880 confocal microscope. Primary antibodies used were rabbit anti-GAD65/67 (Millipore, USA, 1:500 dilution), rabbit anti-VGLUT1 (Millipore, USA, 1:500 dilution), mouse anti-NeuN (Millipore, USA, 1:500 dilution), and mouse anti-c-Fos (Abcam, UK, 1:500 dilution) while secondary antibodies (Thermo Fisher Scientific, USA, 1:500 dilution) were goat anti-rabbit Alexa Fluor 488, goat anti-rabbit Alexa Fluor 568, goat anti-mouse Alexa Fluor 488, and goat anti-mouse Alexa Fluor 568, respectively.

### Electrophysiology

Whole-cell patch-clamp recording was performed in the hippocampal DG region as described previously [109, 110]. P56 male mice were anesthetized with isoflurane and decapitated. Mouse brains were dissected and immersed in ice-cold cutting solution saturated with 95% O_2_ and 5% CO_2_. The cutting solution contained 30 mM NaCl, 26 mM NaHCO_3_, 10 mM D-glucose, 4.5 mM KCl, 1.2 mM NaH_2_PO_4_, 1 mM MgCl_2_, 194 mM sucrose. Coronal brain sections (300 μm) containing the hippocampus (bregma −1.22 to −2.80 mm) were made on a vibrating microtome (Leica Systems, Germany). Slices were allowed to recover for 30 min at 34 °C in artificial cerebrospinal fluid (ACSF) containing 124 mM NaCl, 26 mM NaHCO_3_, 10 mM D-glucose, 4.5 mM KCl, 1.2 mM NaH_2_PO_4_, 1 mM MgCl_2_, 2 mM CaCl_2_, and saturated with 95% O_2_ and 5% CO_2_. Brain slices were then transferred to a holding chamber and stored at RT. Recovery was started at least 1 h before recording. For recordings, brain slices were placed in a recording chamber (Warner Instruments, USA) mounted on an upright microscope (Olympus, Japan) and perfused with ACSF at a rate of 2 mL/min. To record sEPSCs and sIPSCs of DG neurons, patch recording pipettes were filled with intracellular solution containing: 125 mM CsMeSO_3_, 5 mM NaCl, 10 mM HEPES (Na^+^ salt), 5 mM QX314, 1.1 mM EGTA, 4 mM ATP (Mg^2+^ salt), and 0.3 mM GTP (Na^+^ salt). Recording electrodes with a resistance of 4-6 MΩ were pulled from borosilicate glass capillaries (1.5 mm outer diameter) using a P1000 electrode puller (Sutter Instrument Co, USA). Whole-cell voltage-clamp recordings were performed on granule cells with robust activity and a strong stereoscopic sense in hippocampal DG region under an optical microscope. Based on the Cl^−^ concentrations of ASCF and pipette solutions, the Cl^−^ equilibrium potential was calculated as −65 mV according to the Nernst equation. Thus, the GABAergic Cl^−^ flow produced an outward and negative current when the patched cells were voltage-clamped at ± 10 mV. The sEPSCs of glutamatergic transmission were eliminated by holding the membrane potential at ± 10 mV. All recordings were performed at RT, with continuous monitoring of the access resistance of each cell to ensure recording from cells with a series resistance less than 30 MΩ.

### Biocytin-labeled neurons and morphological analysis

To visualize the intricate dendritic branches and dendritic spines within granule cells of the DG in the hippocampus, we employed established biomarkers introduced into intracellular solutions for precise labeling [111]. Horizontal brain slices of the hippocampus were obtained from male mice aged P56-P70. The mice were deeply anesthetized using tribromoethanol (250 mg/kg, ip) and subsequently perfused through the ascending aorta with ice-cold sucrose-based ACSF (sucrose-ACSF) that was preoxygenated (95% O_2_ and 5% CO_2_). The sucrose-ACSF composition was as follows: 220 mM sucrose, 2.5 mM KCl, 1.25 mM NaH_2_PO_4_, 0.5 mM CaCl_2_, 3.5 mM MgSO_4_, 25 mM NaHCO_3_, 20 mM glucoses, 0.4 mM ascorbic acid, and 2 mM sodium pyruvate. This ensured the removal of blood cells and minimized tissue temperature to preserve neuronal integrity and reduce potential fluorescence artifacts. The methodology closely mirrored conventional electrophysiological recording techniques. The recording electrodes, characterized by a higher resistance range of 8-12MΩ, were filled with an internal solution containing: 130 mM K-gluconate, 5 mM KCl, 5 mM phosphocreatine, 10 mM HEPES, 0.5 mM EGTA, 1 mM Na_2_-ATP, 0.3 mM Na-GTP, 2 mM MgSO_4_, and 5 mM biocytin (pH 7.20-7.30, 290 mOsm). The use of electrodes with smaller tips minimized cell damage and facilitated resealing post-recording. Upon cell-sealing, biocytin diffused from the micropipette into the recorded neurons, ensuring comprehensive labeling of complex neuronal structures, including axons and dendritic spines, through a recording duration of 30-40 min. Post-diffusion, the electrode was slowly retracted to reseal the neuron, and excess biocytin was eliminated from the extracellular space by immersing the brain slices in recording solution for 10 min. Subsequently, brain slices were fixed at 4 °C using 4% PFA and rotated on a shaker for 12-24 h. Following fixation, the slices underwent a series of washes in PBS and were then incubated in a solution of PBS containing 0.3% Triton-X100 and 3% goat serum at RT for 2-4 h to permeabilize and block the tissue. Brain slices were incubated in a PBS solution containing 0.3% Triton-X100, 3% goat serum, and 0.1% Fluor 488-streptavidin conjugate (AAT Bioquest Inc., USA) stock solution (1 mg/mL) at 4 °C for 12-24 h. Following three PBS washes, the brain sections were mounted on a microscope slide. All procedures were conducted in darkness on a rotating shaker.

Z-stack images of fluorescently labeled neurons were acquired using confocal microscopy (IXplore SpinSR, Olympus, Japan) with a 60×1.50 NA oil-immersion objective lens. Semi-manual 3D neuron reconstructions were performed using Neuromantic Software [112], initiating from the soma and extending to the distal dendrites. The analysis encompassed determining dendritic intersections at varying distances from the soma using ImageJ (Bethesda, MD, USA, https://fiji.sc/) and assessing dendritic spine density. Neuronal dendritic bifurcation complexity was evaluated via Sholl analysis, while dendritic spine density was manually examined.

### Golgi-Cox staining

The Hito Golgi-Cox kit (Hitobiotec Corp., Wilmington, DE, USA) was employed to assess the structure and density of dendritic spines in hippocampal granule cells. Following euthanasia of the mouse, brain tissue was promptly extracted and cold-washed with 1× PBS for 2-3 s. The brain was then immersed in a mixture of Solution 1 and Solution 2 for 24 h, followed by replacement with a fresh mixture and storage at RT, shielded from light, for 2 weeks. Subsequently, the brain was transferred to Solution 3 and stored for 72 h. Mouse brains were embedded in OCT, and 200-µm-thick brain slices were mounted on gelatin-coated slides, left undisturbed for 24 h in the absence of light before the staining process commenced. After being soaked twice in double-steamed water for 3 min each, the slices were placed in a 1:1 mixture of Solution 4 and Solution 5 for 10 min, followed by another double-steamed water soak for 4 min each time. The subsequent steps involved a gradient dehydration process with 50%, 75%, 95%, and 100% ethanol. Finally, the xylene-cleared brain pieces were encased in resin. Upon drying, the slides were photographed using a bright-field microscope.

### Statistical analysis

GraphPad Prism 8.0 software was used to perform statistical analysis and generate statistical graphs for behavioral and molecular experiments. The electrophysiological statistics were obtained using OriginPro 8.0. The Kolmogorov-Smirnov test were used to analyze the frequency and amplitude of sEPSCs and sIPSCs in cells with Rs < 30MΩ. Student’s *t* test and two-way ANOVA were used to analyze the differences between the two groups. Multiple comparisons were performed using one-way or two-way ANOVA with Holm-Sidak post hoc analysis. Data are presented as mean ± SEM, and *p* < 0.05 was considered statistically significant.

## Supplementary Information

**Additional file 1: Figure S1.** 16p11.2${}^{+/-}$ mice showed gut metabolites disturbance in feces. Changes in the relative abundance of differential metabolites. A Indole-3-propionic acid (IPA). B Adenosine. C Isobutyric acid. D 15-keto Prostaglandin E1 (15-keto-PGE1). E sn-Glycerol 3-phosphoethanolamine (DOPE). F 3-Phenylpropanoic acid. G Ketoisocaproic acid. H Perseitol. I D-Mannitol. J N-Acetyl-L-glutamate. K Scytalone. L N-Acetyl-L-Histidine. M 5-hydroperoxy-6,8,11,14-eicosatetraenoicacid (5(S)-HpETE). N Erucic acid. O 3-Hydroxycapric acid. P N-Oleoylethanolamine. Q Linoleoyl ethanolamide. R Vanillin. S Deoxyadenosine. T Uridine. (WT: *n* = 7 mice; 16p11.2: *n* = 9 mice. Student’s *t* test). Data are presented as mean ± SEM. *p < 0.05 and **p < 0.01. Detailed statistical information is presented in Additional file 2: Table S1. **Figure S2.** 16p11.2${}^{+/-}$ mice displayed hyperactivity but no significant anxiety. A, B 16p11.2${}^{+/-}$ mice showed hyperactivity in the open field test (OFT). C, D 16p11.2${}^{+/-}$ mice showed no significant anxiety-like behavior in the elevated plus-maze (EPM) test (WT: *n* = 11 mice; 16p11.2${}^{+/-}$ : *n* = 10 mice. Student’s *t* test). Data are presented as mean ± SEM. *p < 0.05 and n.s.: not significant. Detailed statistical information is presented in Additional file 2: Table S1. **Figure S3.** 16p11.2${}^{+/-}$ mice exhibited normal activation level in the mPFC and CA1, CA3 of hippocampus. A The red puncta indicate c-Fos^+^ neurons in the CA1 of hippocampus. Scale bar: 50μm. B The density of c-Fos^+^ neurons were not changed significantly in CA1 region of 16p11.2${}^{+/-}$ mice hippocampus (WT: *n* = 5 mice; 16p11.2${}^{+/-}$ : *n* = 4 mice. Student’s *t* test). C The red puncta indicate c-Fos^+^ neurons in the CA3 of hippocampus. D The number of c-Fos^+^ neurons in CA3 of hippocampal region had no distinct difference between the WT and 16p11.2${}^{+/-}$ groups (WT: *n* = 5 mice; 16p11.2${}^{+/-}$: *n* = 4 mice. Student’s *t* test). E The red puncta indicate c-Fos^+^ neurons in mPFC. F No significant changes of the number of c-Fos^+^ neurons in mPFC (WT: *n* = 4 mice; 16p11.2${}^{+/-}$: *n* = 3 mice. Student’s *t* test). Data are presented as mean ± SEM. n.s.: not significant. Detailed statistical information is presented in Additional file 2: Table S1. **Figure S4.** The 16p11.2${}^{+/-}$ mice did not show significant changes in either sEPSC frequency or amplitude, but the decay time and rise time of sIPSC were increased significantly. A Representative sEPSCs traces from granule cells in hippocampus of mice. Scale bars: 5 s, 10 pA. B Cumulative distribution of sEPSCs amplitudes. C. Cumulative distribution of sEPSCs frequencies (WT: *n* = 653 events from 9 cells of 5 mice; 16p11.2${}^{+/-}$: *n* = 281 events from 7 cells of 6 mice. Kolmogorov-Smirnov test). Data are presented as mean ± SEM. Detailed statistical information is presented in Additional file 2: Table S1. **Figure S5.** The effect of IPA on social interaction in diverse behavioral tests. A IPA did not affect the preference of mice for stranger mouse (S1) or empty cage (E) in the sociability phase of TCT (WT+Vehicle: *n* = 20 mice; WT+IPA: *n* = 20 mice; 16p11.2${}^{+/-}$+Vehicle: *n* = 18 mice; 16p11.2${}^{+/-}$+IPA: *n* = 18 mice. Two-way ANOVA). B There was no significant difference in preference between two identical objects (Obj1, Obj2) in the habituation phase of NOR test (WT+Vehicle: *n* = 20 mice; WT+IPA: *n* = 20 mice; 16p11.2${}^{+/-}$+Vehicle: *n* = 18 mice; 16p11.2${}^{+/-}$+IPA: *n* = 18 mice. Two-way ANOVA). C Schematic diagram of USVs behavioral tests in male-female social interaction. D The total number of USVs in adult male mice during the first phase (female present) of the experiment, with or without IPA treatment. E The total number of USVs in adult male mice during Phase 2 (female removed). F The total number of USVs in adult male mice during Phase 3 (female returned) (WT+Vehicle: *n* = 10 mice; WT+IPA: *n* = 10 mice; 16p11.2${}^{+/-}$+Vehicle: *n* = 8 mice; 16p11.2${}^{+/-}$+IPA: *n* = 10 mice). Data are presented as mean ± SEM. *p < 0.05, ****p < 0.0001 and n.s.: not significant. Detailed statistical information is presented in Additional file 2: Table S1. **Figure S6.** Neurobiotin-labeling experiment showed that dendritic branching or the number of dendritic spines of granule cells in DG region was not affected by mouse genotypes or IPA administration. A, B Representative confocal images of dendritic branching (A) and quantitative analysis (B) (WT+Vehicle: *n* = 5 cells from 3 mice; WT+IPA: *n* = 12 cells from 4 mice; 16p11.2${}^{+/-}$+Vehicle: *n* = 14 cells from 4 mice; 16p11.2${}^{+/-}$+IPA: *n* = 7 cells from 3 mice). Scale bar: 50μm. C, D Representative confocal images of dendritic spines (C) and statistical results (D) showed no significant change in the density of dendritic spines in 16p11.2${}^{+/-}$ mice, and IPA had no effect on the density of dendritic spines (WT+Vehicle: *n* = 10 cells from 5 mice; WT+IPA: *n* = 11 cells from 4 mice; 16p11.2${}^{+/-}$+Vehicle: *n* = 14 cells from 4 mice; 16p11.2${}^{+/-}$+IPA: *n* = 8 cells from 3 mice.). Scale bar: 10μm. Data are presented as mean ± SEM. n.s.: not significant. Detailed statistical information is presented in Additional file 2: Table S1. **Figure S7.** Golgi staining showed that the density of dendritic spines of granule cells in DG region was not affected by mouse genotypes and IPA administration. A Representative images of dendritic spines. Scale bar: 10μm. B There was no significant change in the density of dendritic spines in 16p11.2${}^{+/-}$ mice, and IPA did not affect the density of dendritic spines (*n* = 8 slices from 4 mice per group). Data are presented as mean ± SEM. n.s.: not significant. Detailed statistical information is presented in Additional file 2: Table S1.

**Additional file 2: Table S1.** Statistical methods and values of all the results.

**Additional file 3: Table S2.** Significantly differentially expressed genes.

**Additional file 4: Table S3.** PPI results.

**Additional file 5: Table S4.** *C*. *sporogenes* Primers.

**Additional file 6: Table S5.** IPA producing-associated genes primers.

## Data Availability

The raw sequence data of 16S rRNA gene sequencing were deposited in the Sequence Read Archive (SRA) at NCBI under Bioproject PRJNA1065165 (SRR27560768-SRR27560783, https://dataview.ncbi.nlm.nih.gov/object/PRJNA1065165). Data on differential metabolites in mouse feces are shown in Additional file 1: Figure S1. All the other data during this study are available from the corresponding author upon reasonable request.

## Abbreviations

ASD: Autism spectrum disorder
CNVs: Chromosomal copy number variations
IPA: Indole-3-propionic acid
CNS: Central nervous system
TCT: Three-chamber test
DSI: Direct social interaction
NOR: Novel object recognition
OFT: Open field test
EPM: Elevated plus-maze
DG: Dentate gyrus
GABA: Gamma-aminobutyric acid
Glu: Glutamate
E-I imbalance: Excitatory-inhibitory imbalance
GI: Gastrointestinal
GM: Gut microbiome
UHPLC: Ultra-high-performance liquid chromatography
ACSF: Artificial cerebrospinal fluid
5-HT: 5-Hydroxytryptamine
mPFC: Medial prefrontal cortex
sEPSCs: Spontaneous excitatory postsynaptic currents
sIPSCs: Spontaneous inhibitory postsynaptic currents
