# Supplementary material for: The gut metabolite indole-3-propionic acid activates ERK1 to restore social function and hippocampal inhibitory synaptic transmission in a 16p11.2 microdeletion mouse model
Source: Microbiome. 2024 Mar 28;12:66. doi: 10.1186/s40168-024-01755-7 (PMC10976717; doi:10.1186/s40168-024-01755-7)
Supplement: Supplementary file 2 — Additional file 1: Figure S1. 16p11.2${}^{+/-}$+/- mice showed gut metabolites disturbance in feces. Changes in the relative abundance of differential metabolites. A Indole-3-propionic acid (IPA). B Adenosine. C Isobutyric acid. D 15-keto Prostaglandin E1 (15-keto-PGE1). E sn-Glycerol 3-phosphoethanolamine (DOPE). F 3-Phenylpropanoic acid. G Ketoisocaproic acid. H Perseitol. I D-Mannitol. J N-Acetyl-L-glutamate. K Scytalone. L N-Acetyl-L-Histidine. M 5-hydroperoxy-6,8,11,14-eicosatetraenoicacid (5(S)-HpETE). N Erucic acid. O 3-Hydroxycapric acid. P N-Oleoylethanolamine. Q Linoleoyl ethanolamide. R Vanillin. S Deoxyadenosine. T Uridine. (WT: n = 7 mice; 16p11.2: n = 9 mice. Student’s t test). Data are presented as mean ± SEM. *p < 0.05 and **p < 0.01. Detailed statistical information is presented in Additional file 2: Table S1. Figure S2. 16p11.2${}^{+/-}$+/- mice displayed hyperactivity but no significant anxiety. A, B 16p11.2${}^{+/-}$+/- mice showed hyperactivity in the open field test (OFT). C, D 16p11.2${}^{+/-}$ [file 40168_2024_1755_MOESM1_ESM.pdf]

Figures and figure legends of supplementary figures

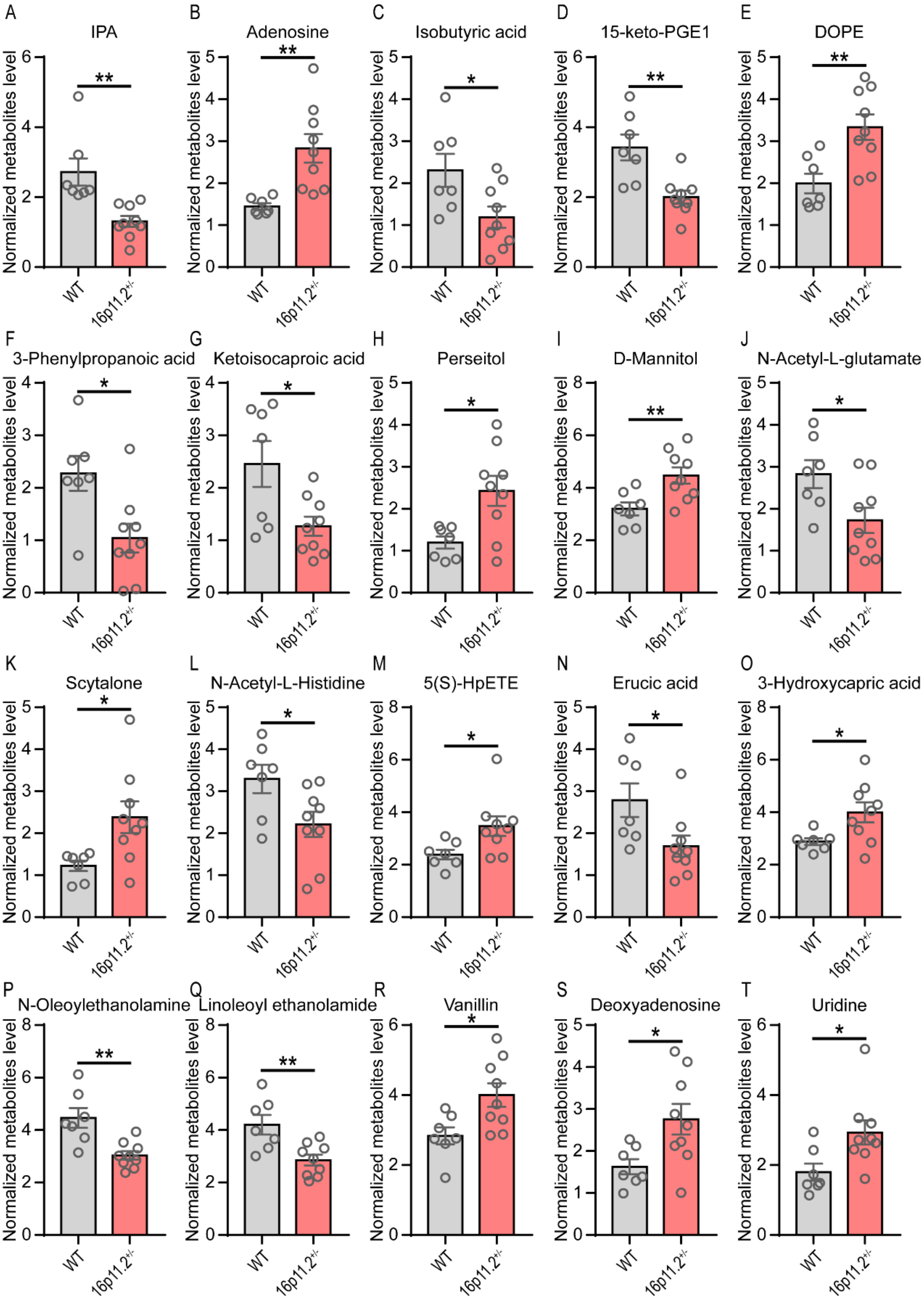

Figure S1 16p11.2$^{+/-}$ mice showed gut metabolites disturbance in feces.

Changes in the relative abundance of differential metabolites. **A** Indole-3-propionic acid (IPA). **B**

Adenosine. **C** Isobutyric acid. **D** 15-keto Prostaglandin E1 (15-keto-PGE1). **E** sn-Glycerol 3-phosphoethanolamine (DOPE). **F** 3-Phenylpropanoic acid. **G** Ketoisocaproic acid. **H** Perseitol. **I** D-Mannitol. **J** N-Acetyl-L-glutamate. **K** Scytalone. **L** N-Acetyl-L-Histidine. **M** 5-hydroperoxy-6,8,11,14-eicosatetraenoicacid (5(S)-HpETE). **N** Erucic acid. **O** 3-Hydroxycapric acid. **P** N-Oleoylethanolamine. **Q** Linoleoyl ethanolamide. **R** Vanillin. **S** Deoxyadenosine. **T** Uridine. (WT: n = 7 mice; 16p11.2$^{+/-}$: n = 9 mice. Student's t-test). Data are presented as mean ± SEM. *$p < 0.05$ and **$p < 0.01$. Detailed statistical information is presented in Additional file 2: Table S1.

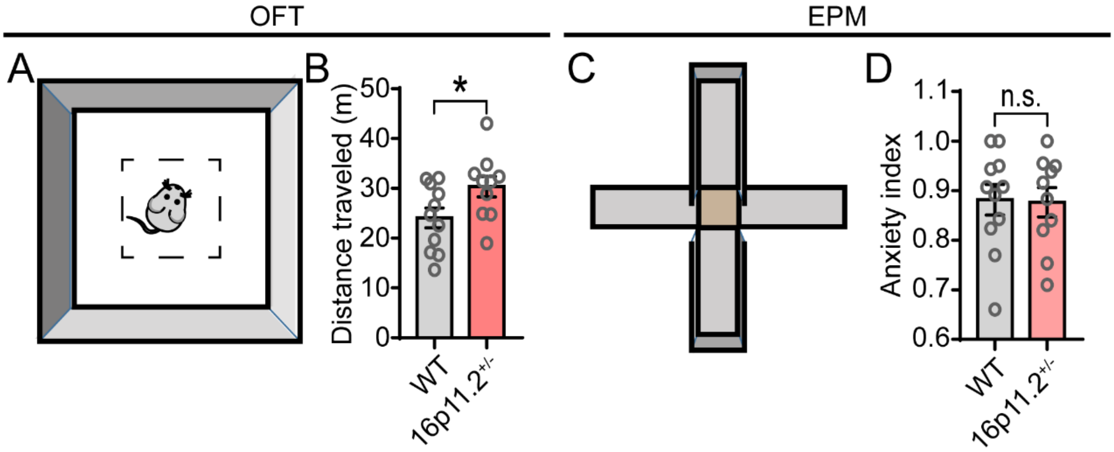

Figure S2 16p11.2$^{+/-}$ mice displayed hyperactivity but no significant anxiety.
A, B 16p11.2$^{+/-}$ mice showed hyperactivity in the open field test (OFT). **C, D** 16p11.2$^{+/-}$ mice showed no significant anxiety-like behavior in the elevated plus-maze (EPM) test (WT: n = 11 mice; 16p11.2$^{+/-}$: n = 10 mice. Student's t-test). Data are presented as mean ± SEM. *$p < 0.05$ and n.s.: not significant. Detailed statistical information is presented in Additional file 2: Table S1.

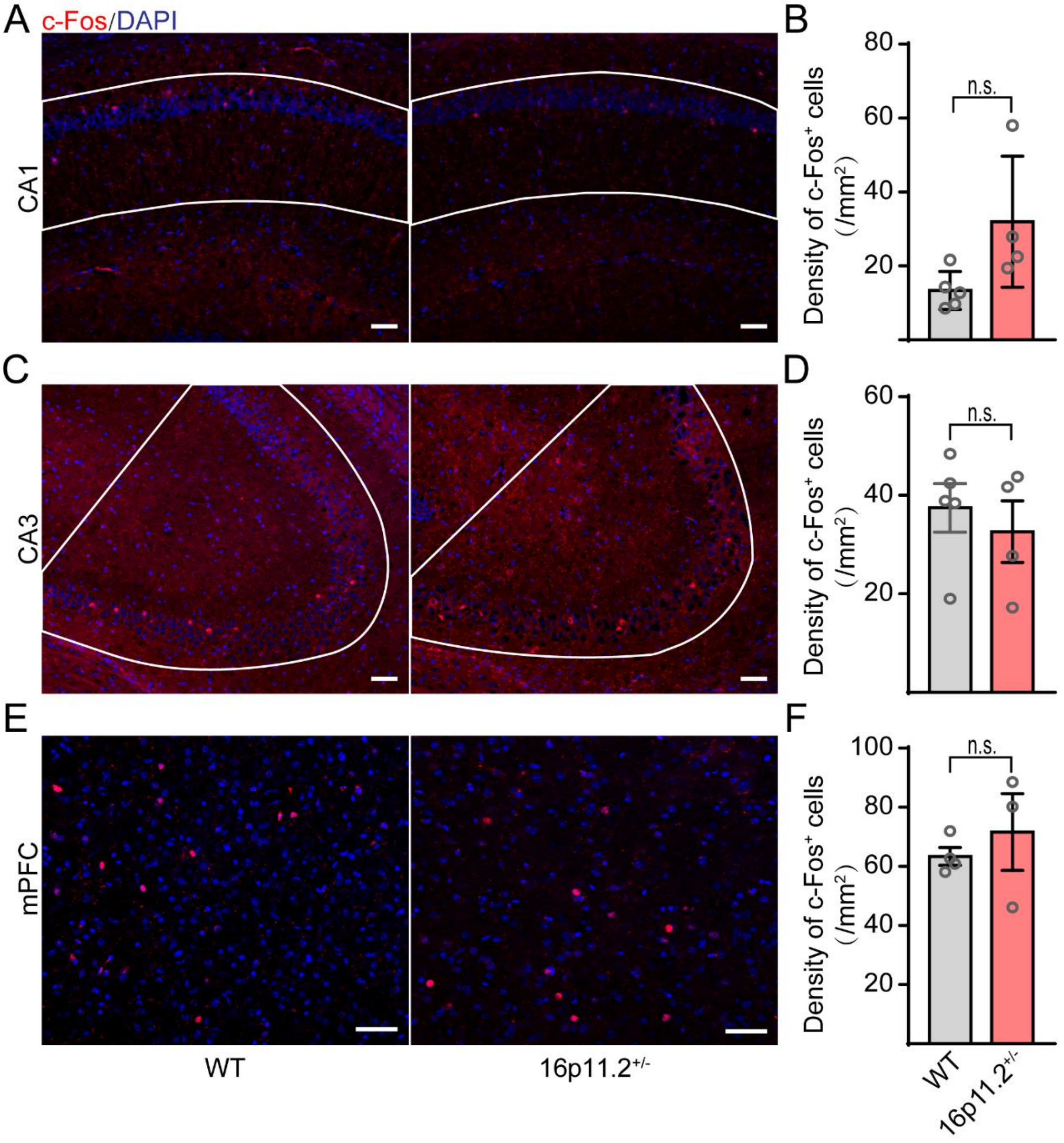

Figure S3 16p11.2$^{+/-}$ mice exhibited normal activation level in the mPFC and CA1, CA3 of hippocampus.

A The red puncta indicate c-Fos^{+} neurons in the CA1 of hippocampus. Scale bar: 50μm. **B** The density of c-Fos^{+} neurons were not changed significantly in CA1 region of 16p11.2$^{+/-}$ mice hippocampus (WT: n = 5 mice; 16p11.2$^{+/-}$: n = 4 mice. Student's t-test). **C** The red puncta indicate c-Fos^{+} neurons in the CA3 of hippocampus. **D** The number of c-Fos^{+} neurons in CA3 of hippocampal region had no distinct difference between the WT and 16p11.2$^{+/-}$ groups (WT: n = 5 mice; 16p11.2$^{+/-}$: n = 4 mice. Student's t-test). **E** The red puncta indicate c-Fos^{+} neurons in mPFC. **F** No significant changes of the number of c-Fos^{+} neurons in mPFC (WT: n = 4 mice; 16p11.2$^{+/-}$: n = 3 mice. Student's t-test). Data are presented as mean ± SEM. n.s.: not significant. Detailed statistical information is presented in Additional file 2: Table S1.

Cumulative probability (sEPSC)

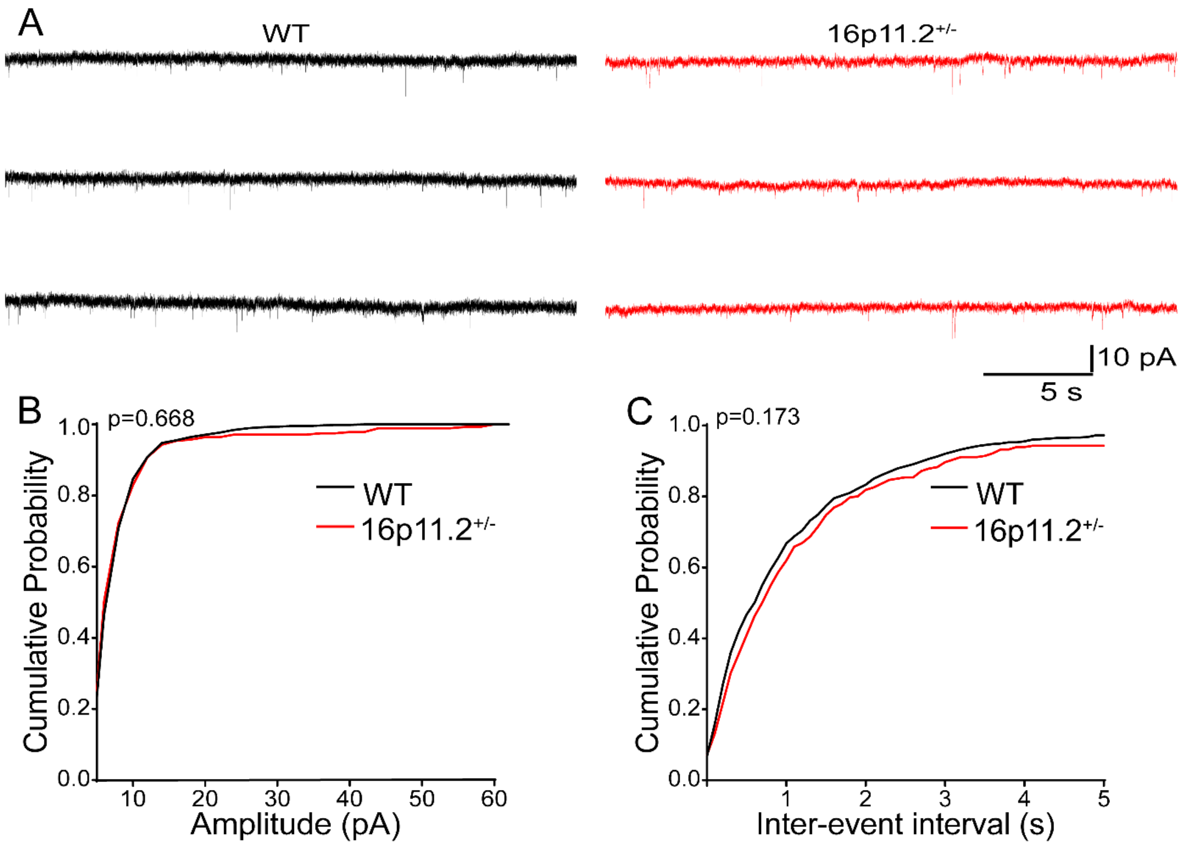

Figure S4 The 16p11.2$^{+/-}$ mice did not show significant changes in either sEPSC frequency or amplitude, but the decay time and rise time of sIPSC were increased significantly.

A Representative sEPSCs traces from granule cells in hippocampus of mice. Scale bars: 5 s, 10 pA. **B** Cumulative distribution of sEPSCs amplitudes. **C** Cumulative distribution of sEPSCs frequencies (WT: n = 653 events from 9 cells of 5 mice; 16p11.2$^{+/-}$: n = 281 events from 7 cells of 6 mice. Kolmogorov-Smirnov test). Data are presented as mean ± SEM. Detailed statistical information is presented in Additional file 2: Table S1.

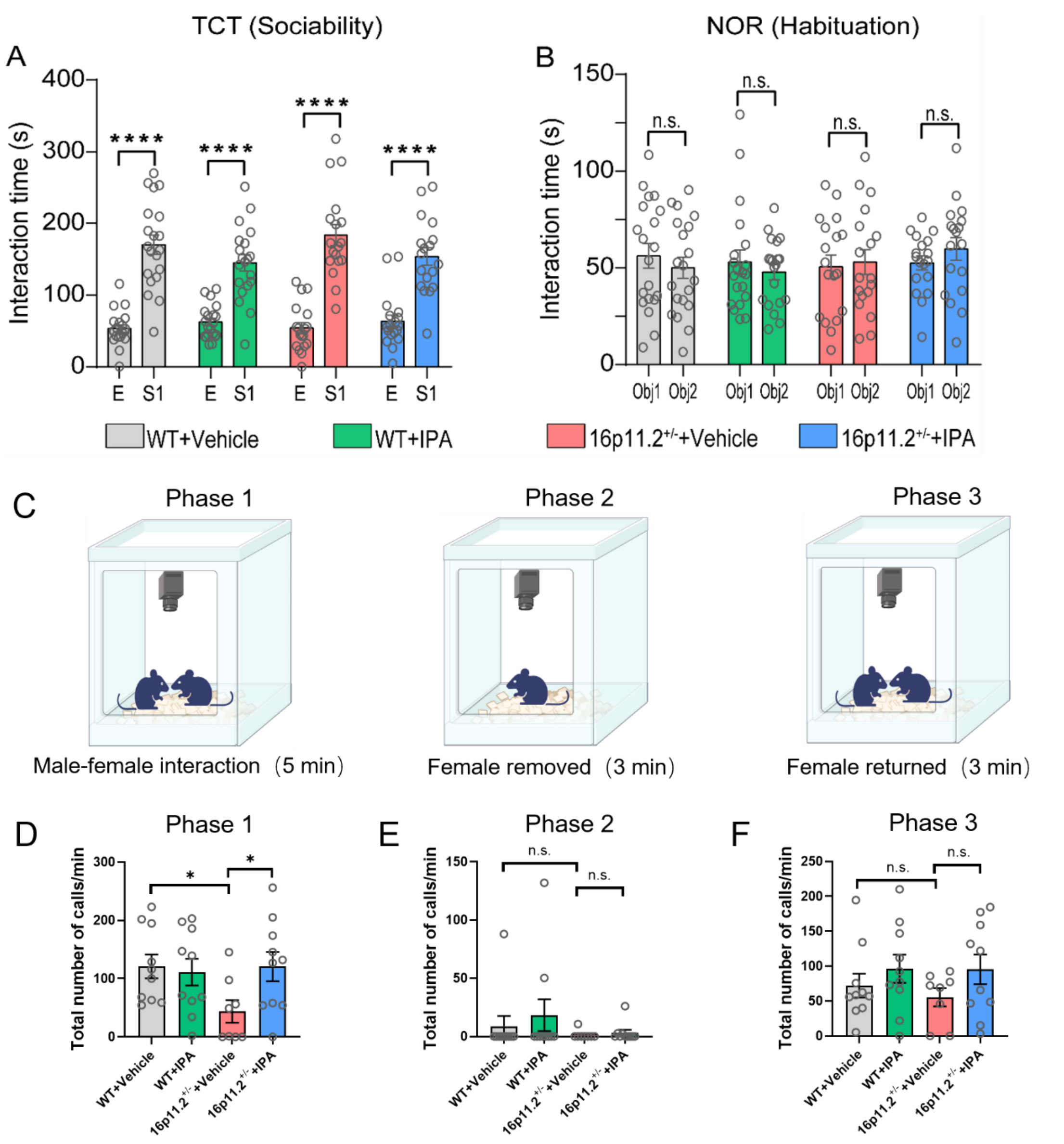

Figure S5 The effect of IPA on social interaction in diverse behavioral tests.

A IPA did not affect the preference of mice for stranger mouse (S1) or empty cage (E) in sociability phase of TCT (WT+Vehicle: $n = 20$ mice; WT+IPA: $n = 20$ mice; 16p11.2$^{+/-}$+Vehicle: $n = 18$ mice; 16p11.2$^{+/-}$+IPA: $n = 18$ mice. Two-way ANOVA). **B** There was no significant difference in preference between two identical objects (Obj1, Obj2) in the habituation phase of NOR test (WT+Vehicle: $n = 20$ mice; WT+IPA: $n = 20$ mice; 16p11.2$^{+/-}$+Vehicle: $n = 18$ mice; 16p11.2$^{+/-}$+IPA: $n = 18$ mice. Two-way ANOVA). **C** Schematic diagram of USVs behavioral tests in male-female social interaction. **D** The total number of USVs in adult male mice during the first phase (female present) of the experiment, with or without IPA treatment. **E** The total number of USVs in adult male mice during the Phase 2 (female removed). **F** The total number of USVs in adult male mice during the Phase 3 (female returned) (WT+Vehicle: $n = 10$ mice; WT+IPA: $n = 10$ mice; 16p11.2$^{+/-}$+Vehicle: $n = 8$ mice;16p11.2$^{+/-}$+IPA: $n = 10$ mice). Data are presented as mean ± SEM. $*p < 0.05$ $****p < 0.0001$, and n.s.: not significant. Detailed statistical information is presented in Additional file 2: Table S1.

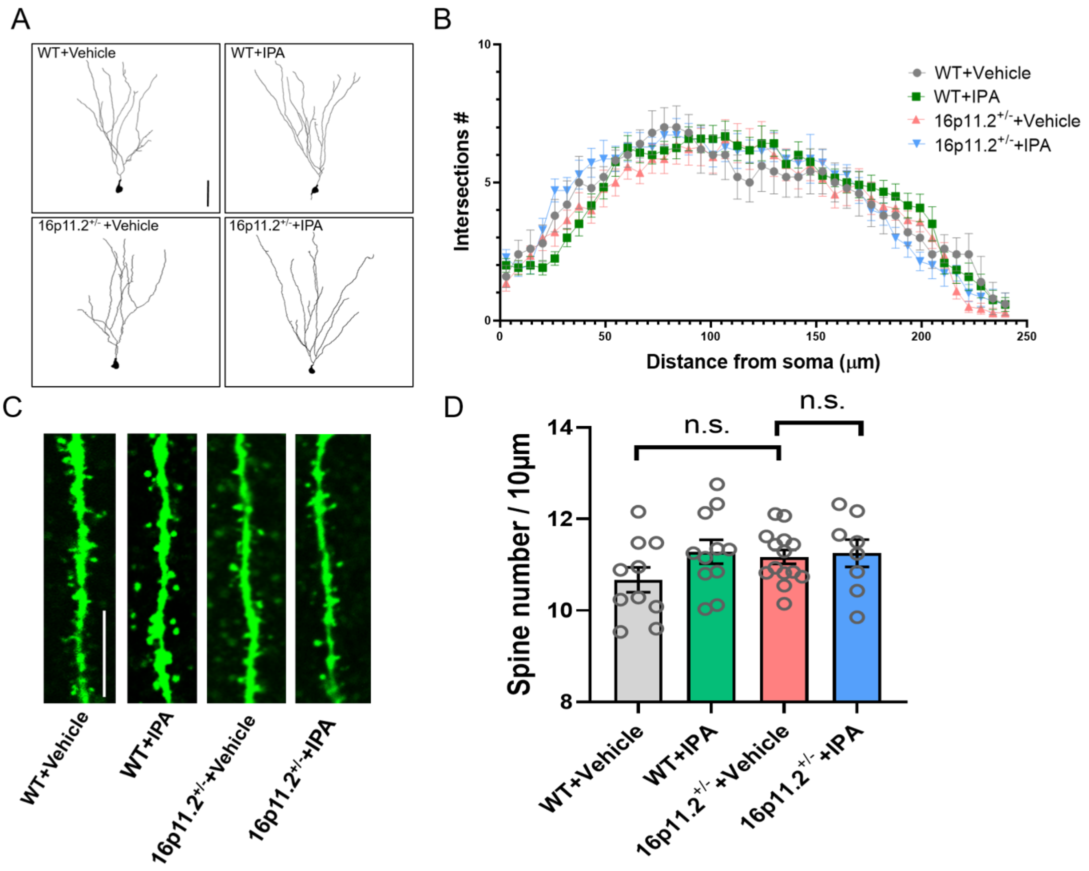

Figure S6 Neurobiotin-labeling experiment showed that dendritic branching or the number of dendritic spines of granule cells in DG region was not affected by mouse genotypes or IPA administration. A, B Representative confocal images of dendritic branching (A) and quantitative analysis (B)，(WT+Vehicle: $n = 5$ cells from 3 mice; WT+IPA: $n = 12$ cells from 4 mice; 16p11.2$^{+/-}$ +Vehicle: $n = 14$ cells from 4 mice; 16p11.2$^{+/-}$+IPA: $n = 7$ cells from 3 mice). Scale bar: 50μm. **C, D** Representative confocal images of dendritic spines (C) and statistical results (D) showed no significant change in the density of dendritic spines in 16p11.2$^{+/-}$ mice, and IPA had no effect on the density of dendritic spines (WT+Vehicle: $n = 10$ cells from 5 mice; WT+IPA: $n = 11$ cells from 4 mice; 16p11.2$^{+/-}$+Vehicle: $n = 14$ cells from 4 mice; 16p11.2$^{+/-}$+IPA: $n = 8$ cells from 3 mice.). Scale bar: 10μm. Data are presented as mean ± SEM. n.s.: not significant. Detailed statistical information is presented in Additional file 2: Table S1.

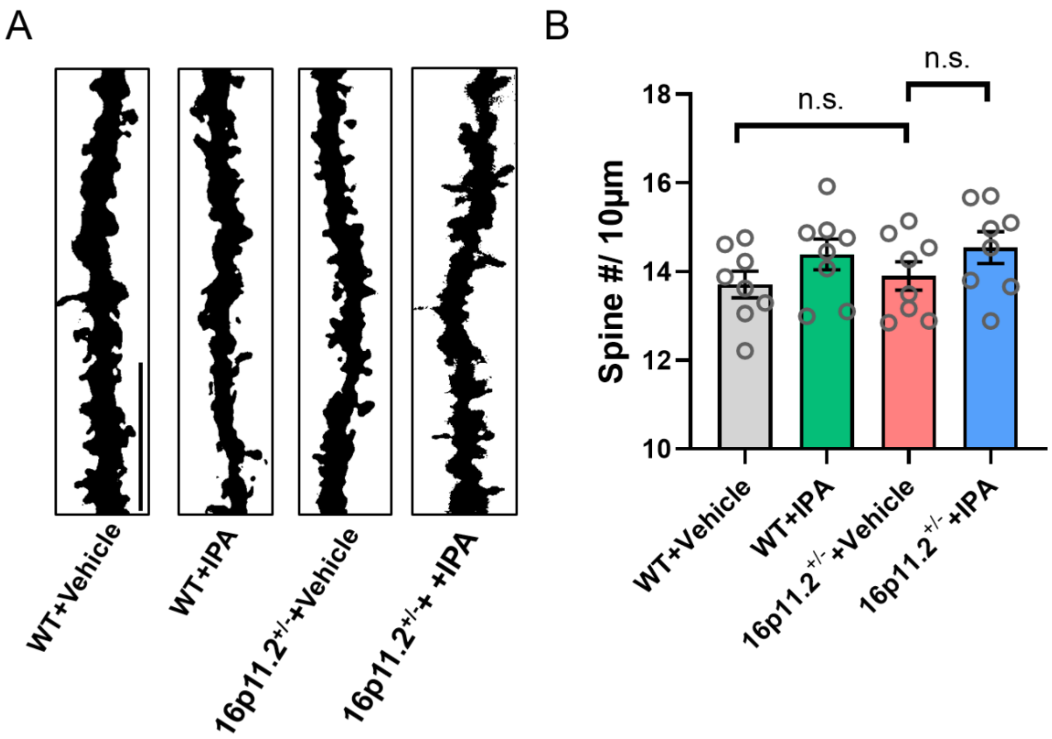

Figure S7 Golgi staining showed that the density of dendritic spines of granule cells in DG region was not affected by mouse genotypes and IPA administration. A Representative images of dendritic spines. Scale bar: 10μm. **B** There was no significant change in the density of dendritic spines in 16p11.2$^{+/-}$ mice, and IPA did not affect the density of dendritic spines (n = 8 slices from 4 mice per group). Data are presented as mean ± SEM. n.s.: not significant. Detailed statistical information is presented in Additional file 2: Table S1.
